# Obtaining new resolutions in carnivore tooth pit morphological analyses: A methodological update for digital taphonomy

**DOI:** 10.1371/journal.pone.0240328

**Published:** 2020-10-08

**Authors:** Lloyd A. Courtenay, Darío Herranz-Rodrigo, Rosa Huguet, Miguel Ángel Maté-González, Diego González-Aguilera, José Yravedra

**Affiliations:** 1 Department of Cartographic and Terrain Engineering, Higher Polytechnic School of Ávila, University of Salamanca, Ávila, Spain; 2 Department of Prehistory, Complutense University, Madrid, Spain; 3 C. A. I. Archaeometry and Archaeological Analysis, Complutense University, Madrid, Spain; 4 Institut Català de Paleoecologia Humana I Evolució Social (IPHES), Tarragona, Spain; 5 Department d’Historia i Historiao de l’Art, Universitat de Rovira I Virgili (URV), Tarragona, Spain; 6 Unit Associated to CSIC, Departamento de Paleobiologia, Museo de Ciencias Naturales, Madrid, Spain; 7 Gran Duque de Alba Institution, Diputación Provincial de Ávila, Ávila, Spain; 8 Department of Topographic and Cartography Engineering, Higher Technical School of Engineers in Topography, Geodesy and Cartography, Technical University of Madrid, Madrid, Spain; The Cyprus Institute, CYPRUS

## Abstract

Modern day investigation in fields of archaeology and palaeontology can be greatly characterised by an exponential growth of integrated new technologies, nevertheless, while these advances are of great significance to multiple lines of research, their evaluation and update over time is equally as important. Here we present an application of inter and intra-observer analysis in taphonomy based geometric morphometrics, employing robust non-parametric statistical analyses for the study of experimental carnivore tooth pit morphologies. To fully understand the influence of measurement errors in the collection of this data, our statistical assessment was performed on fully superimposed, partially superimposed and raw landmark coordinates collected from 3D surface scanning. Experimental samples used to assess these errors includes wolf and dog tooth pits used in modern day ecological livestock predation analysis. Results obtained from this study highlight the importance of landmark type in the assessment of error, emphasising the value of semi-landmark models over the use of ambiguous Type III landmarks. In addition to this, data also reveals the importance of observer experience for the collection of data alongside an interesting increase in error when working with fully superimposed landmarks due to the “Pinocchio Effect”. Through this study we are able to redefine the geometric morphometric models used for tooth pit morphological analyses. This final hybrid Type II fixed landmark and semi-landmark model presents a significant reduction in human induced error, generating a more metrically reliable and replicable method that can be used for data pooling in future inter-institutional research. These results can be considered a fundamental step forward for carnivore inspired studies, having an impact on archaeological, palaeontological, modern-day ecological research as well as applications in other forensic sciences.

## 1. Introduction

The identification and analysis of the Bone Surface Modifications (BSMs) produced by different taphonomic agents is a fundamental component in modern archaeological, palaeontological and, more recently fields of applied forensic sciences. Among the different types of BSMs, in prehistory tooth marks present valuable information that greatly contribute to the understanding of site formation processes, palaeolandscapes and their associated ecologies. Under this premise, many authors have paid particular attention to the different tooth marks that different animals may produce, whether this be for the consumption of meat or other practices of biological origin such as osteophagia or general foraging activities.

Among tooth mark studies, mammalian carnivores are a particularly important protagonist in archaeological research [1–5], considering their competition for resources with hominins throughout the Pleistocene. Interestingly herbivores [6–8], omnivores [9, 10], insects [11], and birds [12, 13], are also known modifiers of osteological remains, causing some confusion in the classification and interpretation of BSMs. Rodents such as the porcupine (*Hystrix sp*.) are an additional well documented bone accumulator [4, 14–16], proving to be problematic agents in the interpretation of faunal accumulations. Likewise, chondrichthyans [17, 18] as well as large [19, 20], and very large reptiles [21–23], are also of great importance especially in paleontological research. Last but not least, human tooth modifications to bone are also a compelling line of investigation with great value in archaeology [24, 25].

Through these studies, current understanding of carnivore BSMs enumerate 4 main types of alterations produced by teeth [1, 5]. These include rounded circular depressions caused by the direct imprint of the cuspid on bone (*pits*); elongated depressions with a circular base produced by the dragging of teeth across the cortical surface (*scores*); circular holes produced by the direct penetration of the tooth through the cortical walls (*puncture*); and the progressive deletion of large portions of bone through continuous chewing (*furrowing*).

To date a great deal of investigation has focused their efforts on the systematic study of pits and scores. This type of research has gone through numerous different phases, starting with a simple qualitative *in-visu* characterisation of these traces [1–5, 17, 18, 22, 23], to a more in depth integration of quantitative analyses [10, 21, 25–29]. In many cases, the use of metric variables such as the length and width of tooth marks have proven valuable variables in the interpretation of archaeological and paleontological sites [19, 30–34, *inter alia*]. Nevertheless, a common limitation noted by most is the ability to go beyond the size of the carnivore chewing and specifically identify the agent(s) present in a site.

New possibilities have recently been made available through Geometric Morphometric Methods (GMM). GMM are a powerful multivariate statistical toolset for morphological analyses [35, 36], originating primarily from fields of biology, evolutionary sciences, systematics, and physical anthropology [37–44]. Outside of anatomy, however, GMM has had other successful integrations into other aspects of archaeology, such as the study of lithic materials [45, 46, *inter alia*].

For geometric morphometric analysis, the identification and quantification of points of interest, known as *landmarks*, are required. These points can be defined as “a point of correspondence on an object that matches between and within populations” [47: pg. 3]. One advantage of landmarks is that they can either be recorded as 2D or 3D coordinates in space, providing a direct means of visualising variations between morphologies through the distortion or displacement of each landmark on each individual [48, 49]. The configuration of these points in space additionally present the advantage of preserving the full geometry of the specimens being studied [37]. Finally with the introduction of powerful 3D modelling equipment and methodological protocols, the capturing of morphological data is much more precise.

In BSM taphonomy, GMM were initially introduced as a means of quantifying cut mark morphologies for both 2D derived data [50] and entire 3D models [51]. These advances have obtained exceptional resolutions, presenting means of identifying the lithic tool and raw material used to produce each cut mark in archaeology [50–52, *inter alia*], while also arising in forensic research for sharp force trauma identification [53, 54, *inter alia*]. Likewise, initial efforts in the quantification of tooth score and pit morphologies reached up to 80% classification rates differentiating between carnivore agencies [55–58], with Machine Learning algorithms obtaining > 95% accuracy [58, 59].

Regardless of this success, however, it is important to consider the value of the landmark models being used for geometric morphometric data collection. To name one primary issue, landmarks can be further divided into three different types; easily locatable landmarks (Type I) and landmarks whose location are more ambiguous (Types II and III). Type I landmarks in biology are usually anatomical features of a precisely defined nature (e.g. tendon insertions), while Type II landmarks are defined by local properties (e.g. maximum curvature) and Type III landmarks are constructed points across the structure (e.g. a centroid) [35, 47: pg. 3–4]. From this concept, each type of landmark possesses a certain degree of error and subjectivity that must be addressed [60, 61]. Possible issues can be extrapolated to include; the experience of the researcher placing the landmark and analyst inconsistencies throughout measuring sessions [62–69]; the natural variability and preservation rate of the landmark itself [62, 70–73]; the landmark’s definition and how easy it is to pinpoint [60–62, 67, 70]; and finally the precision of the measuring device being used [67–69].

Similarly, variability according to inter- and intra-observer error additionally has a significant effect on the comparison of information produced in different studies [67]. The ability to share data is a fundamental component of modern day research [74], especially in areas where accessibility to certain types of data is costly. This is a considerable issue when considering data derived from carnivore feeding, where in many cases large sample sizes are hard to obtain or access to wild animals may not be easy for researchers who reside on different continents [75]. Moreover, special care must be taken when working with animals, considering the conditions required to ensure correct welfare practices [76]. Under this premise, the ability to share data must be considered fundamental in order for research to advance.

While some initial approximations have been made to assess the reliability of geometric morphometric models in carnivore BSM analyses [55: pg. 97, 57: S2 Appendix], an in-depth study is yet to be carried out. For example, the current model for tooth pit analyses is known to produce highly complex dataset of high dimensionality. One particular issue with these datasets also includes the low proportion of cumulative variability represented by each variable [59]. Moreover, tooth pit models are based primarily on Type III landmarks, some of which are questionably much harder to locate than others. To ensure the reliability of this methodological approach, and where possible improve its precision, a detailed assessment is necessary.

The present study thus demonstrates how a detailed revision and update of Aramendi et al. [55]’s original model can facilitate future analyses of carnivore tooth pits with greater statistical reliability and accuracy. This in turn can be considered an important obstacle to supersede in order to make carnivore tooth mark data readily available to other researchers. Such advances are of great value to the study of carnivore BSMs; applicable to archaeological, palaeontological and modern day ecological research.

## 2. Methods

### 2.1. Tooth mark samples and digitisation

A total of 60 carnivore tooth pits were studied and compared for the purpose of this study. All marks were collected on the long bone diaphyses of large sized animals, including tibiae and radii. Diaphyses were chosen considering their greater density than epiphyses and are thus more likely to survive during carnivore feeding. Samples were produced in a controlled setting and include tooth marks produced by *Canis lupus signatus* wolves (pits = 30) and an Irish Setter gundog (pits = 30). Tooth mark samples from both carnivores were obtained in a controlled setting during the feeding of these animals. For the case of wolves, these samples were obtained from semi-captive animals (>1000m^2^) from the Cabárceno natural park (Cantabria, Spain, http://www.parquedecabarceno.com) following standard feeding protocol established by the park. Wolves were left with the bones for 1 week before the samples were recollected by park employees. In the case of gundog tooth samples, bones were provided using product obtained from a local butcher. In this case, bones were left with the dogs for less than a day in an equally controlled environment. All samples were donated for use in the study, and no permits were required for their collection. Once collected, if necessary bones were cleaned in boiling water prior to analysis.

Each of these samples were originally studied by Yravedra et al. [58], identifying significant differences in tooth pit morphology for the identification of livestock predation in Europe. These bone surface modifications can thus be used as a control sample considering how morphological variations are already known in detail.

**Fig 1:**
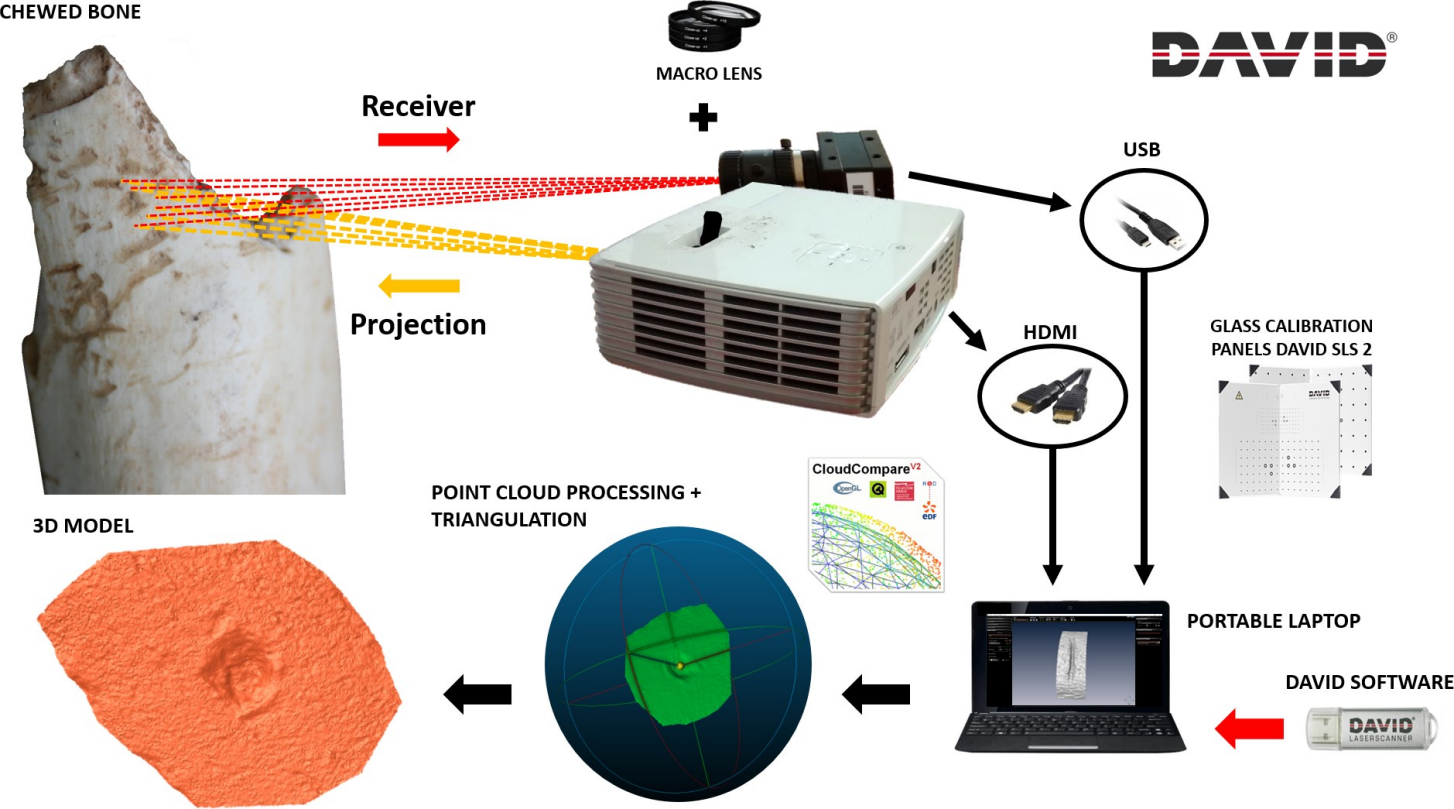
A graphical description of the digital reconstruction protocol used in the present study; producing 3D models of carnivore tooth marks on bone according to Maté-González et al. [77].

Digital reconstructions (Fig 1) of each mark were performed using the DAVID SLS-2 Structured Light Surface Scanner located at the TIDOP Research Group of the Higher Polytechnic School of Ávila (University of Salamanca, Spain). Equipment was calibrated using a 15 mm marker board while both the projector and the camera were equipped with additional macro-lenses (x2 to x10) for optimal resolution at microscopic scales [77]. Digitisations using this equipment are carried out in ca. 1 minute producing a point cloud density of up to 1.2 million points.

### 2.2. Geometric morphometric data collection

For the purpose of this study, landmark data collection was performed by three separate individuals (A1-3) with varying degrees of experience. The first analyst (A1) is an experienced taphonomist, familiar with the different types of bone surface modifications produced by carnivores, yet has no prior experience working with geometric morphometric data. This analyst was chosen as a true analogy of a researcher in taphonomy who may be interested in applying GMM to their own analyses. The second analyst (A2), on the other hand, is a specialist in remote sensing and the handling of three-dimensional data, having worked in the past with geometric morphometric data. Finally, the third analyst (A3) is both a specialist in taphonomy and has the greatest extent of experience working with geometric morphometric data.

**Fig 2:**
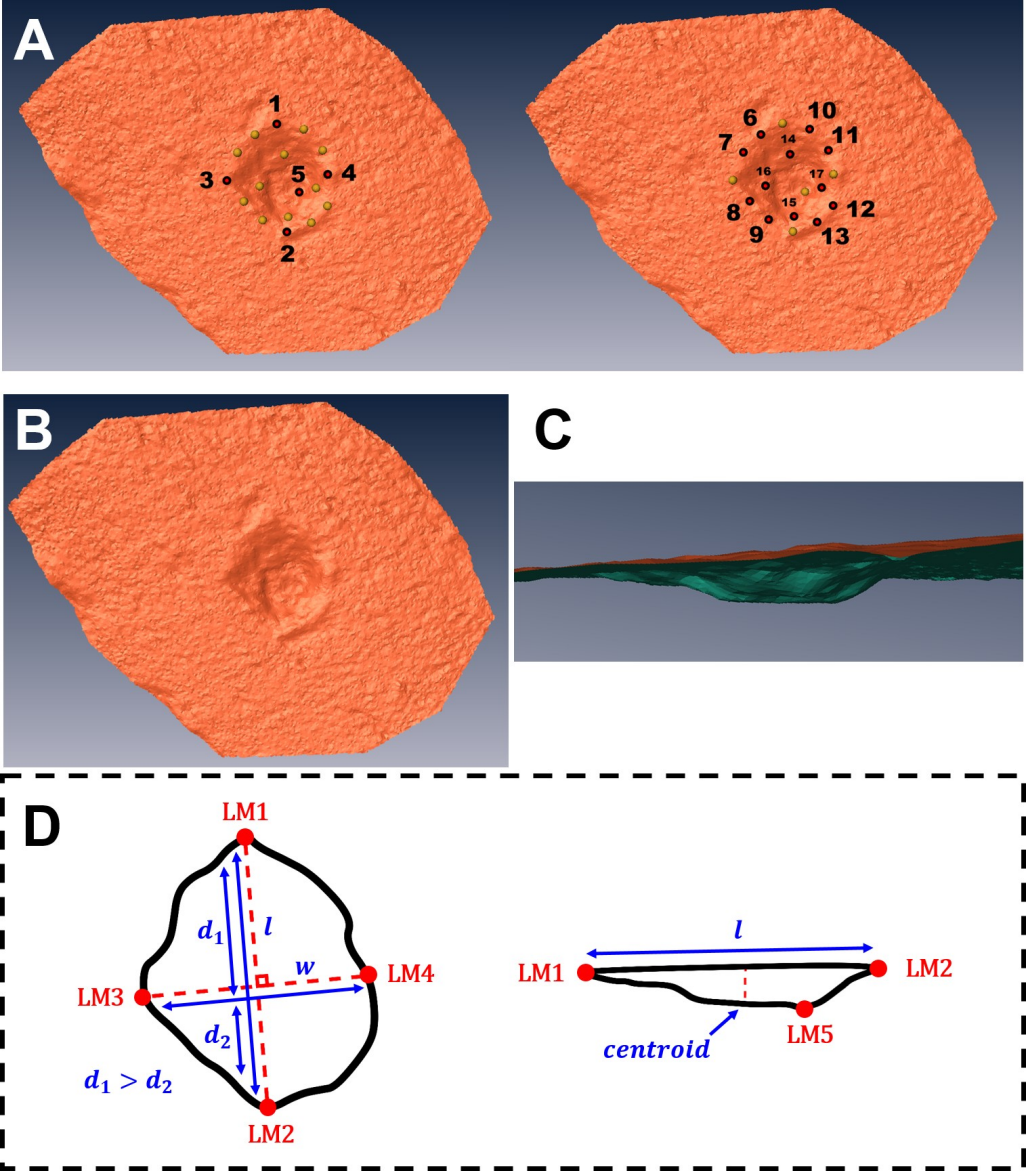
A detailed graphical description of the landmark models employed within this study. (A) The original 17-Landmark model [51], placed on the pit presented in (B) and (C). (D) The detailed description of the precise locations of landmarks 1 to 5 in the revised version of this model.

The main landmark configuration used for this study was that proposed by Aramendi et al. [55], consisting in a mixture of 17 fixed Type II and Type III 3D landmark points on the exterior and interior surfaces of each pit (Fig 2A). Each of the analysts performed collection of landmark data in separate locations, all using the free Landmark Editor software v. 3.0.0.6 [78]. Before data collection began, each of the participants were provided with the original paper [55] describing the landmark configuration to be used, alongside some general instructions on how to use the software. Throughout the process, none of the participants were allowed contact during the procedure so as to avoid influencing their placement of points.

To ensure an identical orientation of the pit by each observer, thus facilitating any consequent analyses, the only additional instruction provided was on how to identify the position of landmark n°1. Landmark n°1 was thus defined as the point along the axis marking the maximum length furthest away from the perpendicular axis marking the maximum width.

Once 3D landmark data had been collected by each of the analysts, coordinates were formatted and imported into R v. 3.5.1. (https://www.r-project.org/) for further statistical processing.

### 2.3. Inter- and intra-observer analyses

As described by Cramon-Taubadel et al. [65], four main approaches exist for the comparison of inter and intra-observer errors. The first, and arguably most common approach, consists in projecting repeated measurements onto a common coordinate system [79]. This is most commonly performed through Generalised Procrustes Analyses (GPA) [41, 79, 80], consisting in a series of superimposition procedures, including scaling, rotation and translation. If after GPA the measured landmarks fall into a suitable range of variation, then the configuration can be considered reliable. The second method employs Euclidean distances between repeat measurements and the centroid of the configuration to assess the relative repeatability of each landmark [38]. The third approach consists in the repeated digitisation of a specimen that is held in a constant orientation, thereby directly assessing the error of a landmark coordinate through variation in landmark location [81]. The final approach consists in the partial superimposition of landmark configurations so as to reduce the impact of the “*Pinocchio effect*” [82], a phenomenon described by the distortion of measurements influenced by scaling procedures in GPA from the first and second approaches [65].

For the purpose of this study, a mixture of these approaches were employed on the analysis of observers studying different carnivore tooth pits. For the first part of these analyses, Euclidean distance calculations between points placed by each analyst were performed prior to any superimposition procedures. This was carried out on the raw coordinates obtained directly from the 3D models, providing a metrically accurate true approximation in millimetres to the margin of error of each landmark [81]. Additional calculations for inter-analyst variability in raw feature space was performed by calculating the Euclidean distance between the point placed by each analyst and the centroid between all three analysts [69].

This was followed by the full superimposition of landmarks through GPA (*shape space*) and the calculation of mean reference configurations for each of the analysts separately, the carnivore samples, and for the entire sample as a whole. Principal Components Analyses (PCA) were then performed to examine the distribution of specimens in morphospace. If error among analysts is low, PCA should present a tight clustering of individuals regardless of the analyst recording the data. From the derived PC scores, Multivariate Analyses of Variance (MANOVA) tests were performed to statistically assess these variances. For MANOVA, either the “Hotelling-Lawley” or “Wilk’s Lambda” formulae were used for homogenously and inhomogeneously distributed coordinates respectively. For homogeneity testing, both numerical tests and Quantile-Quantile (Q-Q) plots were used on landmark point clouds, PCA results and distance calculations to avoid issues induced by sample size [83].

From the fully imposed feature space, Procrustes distances were also calculated between a reference shape and the configuration of landmarks placed by each of the analysts. Here the mean configuration of all individuals was used as the reference shape. From these Procrustes distances, further statistical tests and metrics were used to evaluate the amount of error and variability produced. While most traditional studies of intra and inter-observer measurement errors represent within-group variance as an estimation of the within-observer mean squares or mean distances [e.g. 63, 66, 67], in this study robust statistical metrics were employed [83], using the Biweight Midvariance (BWMV) (Eqs 2–4), calculated via the median of errors and their consequent Median Absolute Deviation (MAD) (Eq 1).

$$MAD=m(|x_{i}-m_{x}|)$$ (1)

$$BWMV=\frac{n\sum_{i=1}^{n}a_{i}{\left(x_{i}-m\right)}^{2}{\left(1-U_{i}^{2}\right)}^{4}}{{\left(\sum_{i=1}^{n}a_{i}\left(1-U_{i}^{2}\right)(1-5U_{i}^{2})\right)}^{2}}$$ (2)

$$a_{i}=\{\begin{array}{cc} 1, & if|U_{i}|<1\\ 0, & if|U_{i}|\geq 1 \end{array}$$ (3)

$$U=\frac{x_{i}-m}{9MAD}$$ (4)

MAD is a calculation of the median (*m*) of the absolute deviations from the data’s median (*m*_*x*_) which is normally reported as a normalized value (NMAD). Normalisation of MAD is calculated through multiplying by the constant 1.4826. This is carried out to provide a scale factor of the true domain’s standard deviation, using a 0.75 percentile as reference (Eq 5). Under this premise, MAD values are scaled by a factor of (Eq 5):

$$\frac{1}{\Phi^{-1}\left(\frac{3}{4}\right)}\approx 1.4826$$ (5)

Where Φ^-1^ is the inverse cumulative distribution function of a standard Gaussian distribution [84].

NMAD has the distinct advantage of being a more robust measurement that is resilient to outliers in a dataset [83]. This is particularly useful for the analysis of datasets that do not follow a Gaussian distribution. The BWMV (reported as its square-root) is a further non-parametric measurement that additionally presents robustness of efficiency, proving a valuable substitute in non-parametric calculations [85]. NMAD and the square-root of the BWMV have proven to be important variables in accuracy control and system assessment analysis in remote sensing applications [83, 85–89]. Because of this, these metrics were employed as a substitute for standard deviation error calculations where Gaussian distributions were not detected, while the central tendency is reported as the median. Nevertheless, where deemed necessary by the normality of the dataset’s distribution, further mean and standard deviation calculations were also reported.

Likewise, while most traditional analyses of observer measurement errors employ the use of Type II Analysis of Variance (ANOVA) calculations or Tukey’s pairwise post-hoc comparisons [63, 64, 66–68, 90, 91, *inter alia*], these tests are statistically and mathematically conditioned by the underlying assumption of normality in the data’s distribution [92, 93]. In cases where distances and errors do not fit a Gaussian distribution, parametric ANOVA tests are unlikely to truly capture the true nature of variances in sample distributions [94]. Because of this, when non-Gaussian distributions were detected, the present study employed the use of an adaptation of ANOVA known as a non-parametric Kruskal-Wallis robust statistical test to assess differences in distribution medians [95; pg. 585, 587 & 598]. This test employs a rank-based H statistic, similar to the more traditional *x*^2^ or F statistic, that is able to “detect the kinds of difference of real interest” while making “only general assumptions… about the kind of distributions from which the observations come” [95; pg. 585].

Further testing considered the use of *Repeatability Measures* (RM); a calculation of the proportion of variance due to true variation among individuals [63, 66]. RMs can be defined as calculations involving the sum of squared distances (*d* ^2^) through (Eq 6):

$$RM=\frac{\left(\frac{1}{n-I}\sum_{j=1}^{J}d_{j}^{2}\right)}{\left(\frac{1}{n-I}\sum_{i=1}^{I}\sum_{j=1}^{J}d_{ij}^{2}\right)}$$ (6)

where *n* is the total number of individuals in all of the samples, *I* is the total number of samples and *J* is the number of individuals in each of the samples. *d* values used to calculate this metric were the Procrustes distance. The RM metric is reported as a value ranging from 0 to 1; the latter indicating variance in measurements being attributable to the analyst under study (A_j_), rather than the true variation within the dataset [63].

From Procrustes distance calculations, further analyses included the generation of Unweighted Pair Group Method with Arithmetic Mean (UPGMA) trees. UPGMA results were analysed according to tree topologies, testing to see whether tooth pits clustered together according to the animal producing the tooth mark or the observer recording landmark data. Neighbour Joining (NJ) tree algorithms were also contemplated, nevertheless in this case NJ proved harder to read and was therefore discarded

Following this, two unsupervised Machine Learning algorithms were trained on fully superimposed coordinate data to test and see whether landmarks could be clearly defined through pattern recognition techniques. These analyses were performed in Python v.3.7 (http://www.python.org), using the SciKit-Learn package. Python applications were additionally implemented into the rest of the R workflow using the reticulate package and the RStudio development environment (https://rstudio.com/).

The first algorithm used was a Density-Based Spatial Clustering Algorithm with Noise (DBSCAN). DBSCAN employs a series of non-parametric mathematical and logical theories that differentiate between points that can be classed as reachable from its clustering core, and those that are considered as outliers or *noise points* ([96]: {*P*ϵ*D*|∀|*i*: *p* ∉ *C*_*i*_ where point *p* in dataset *D* is not associable to any of the clusters *C*). DBSCAN requires the definition of 2 hyperparameters; the ε value defining the neighbourhood of a point and the minimum number of points (MinPts) that are required to form a cluster (i.e. the density of points). MinPts was set to 30, considering each of the analysts were required to digitise 30 individuals per sample for this study. *ε* values were established in accordance with both the corresponding MinPts value, as well as the nature of the dataset’s distribution. This value can be objectively defined mathematically using k-distance graphs according to the nearest neighbour [96], while the “elbow-joint” method can be used to establish the optimal *ε* value for a dataset through calculating the point of maximum curvature in the plotted *k*-distance graph. The final *ε* value for this study was thus calculated at 0.04 using the “kneed” algorithm in Python [97].

The second pattern recognition algorithm used was the non-parametric Mean-Shift algorithm (MS). MS, much like DBSCAN, works through calculating areas of high point density with the use of a kernel function, also allowing for the mathematical detection of outliers [98–100]. MS only requires the definition a single hyperparameter, known as the bandwidth, which is used to define the size scale of the algorithm’s internal kernel function. In this study, the bandwidth was established using Scikit-Learn’s bandwidth estimation functions, employing a calculated quantile value of 0.05 to fine tune this estimation.

The objectives of using pattern recognition algorithms consisted in defining the degree of separation between landmarks. Landmarks that can be clearly defined by all three analysts should theoretically appear clearly differentiable by both algorithms with as little noise as possible. The results produced by these algorithms were evaluated by considering the number of identified clusters, the number of noise points, and the number of points that were correctly or incorrectly grouped with other points within each cluster.

The final part of this analysis performed calculations on partially superimposed landmarks, carried out by removing the scaling procedure from GPA (*form space*). This ensures that each of the configurations are centred and superimposed without creating statistical noise from the scaling process and thus factoring in the possible distortion that could be created by the “*Pinocchio effect*” in GMM [65, 82]. On partially superimposed data, PCA and Procrustes distance calculations were performed. From these distances, UPGMA trees were additionally calculated.

### 2.4. Semilandmark models

Once the accuracy and reliability of each landmark had been assessed, trials were performed removing landmarks most prone to inter and intra-analyst error and replacing them with semilandmarks (S1 Appendix) [35, 73, 101]. Semilandmarks are computed equidistant points that slide along the surface of the 3D model [73]. This overcomes many issues in accuracy when dealing with features that can only be represented by Type III fixed landmarks. Considering these landmarks are most prone to error and often incorrectly placed [61], substituting these points with semilandmarks presents a more efficient means of objectively quantifying complex morphological features such as curves and irregular surfaces [68].

For the process of placement of these semilandmarks, patches were drawn over each pit in the Landmark Editor v.3.0.0.6 software and different numbers of semilandmarks were computed. Trials were performed to find the optimal number of semilandmarks for tooth pit analysis, including 5x5, 6x6, 7x7, 8x8, 9x9 and 10x10 configurations.

The final part of this study consisted in both a full and partial GPA of the final landmark data to assess the variances in shape and form space captured by the newly updated landmark configurations. PCA were then performed to represent these differences graphically and to reduce the dimensionality of the dataset for further multivariate statistical processing. Comparison of PCA results reported the dimensionality of the consequent feature space (ℝ^n^), the proportion of variance represented by the first 2 PC scores as well as the cumulative proportion of variance represented by the first 10 PC scores. These were followed by MANOVA calculations to assess the degree of separation between wolf and dog samples according to the landmark configuration used. Finally, a Canonical Variance Analysis (CVA) was performed, alongside calculations of Mahalanobis and Procrustes distances and permuted (n = 999) *p*-values to evaluate the degree of separation between the samples.

## 3. Results

### 3.1. Inter- and intra-observer errors

Throughout the study, clear patterns emerged indicating those landmarks that were easier to locate and those that were more difficult. In all three trials, A1-3 noted difficulties on locating Landmarks (LM) 14 through to 17, while LM1-5 were considered the most straightforward. A1-3 also noted that LM6-13 were easy to locate once LM1-4 had been clearly established. Nevertheless, *in-visu* inspection of each of the measurements taken on the 3D models documented that some analysts were more meticulous upon placing these points than others. Interestingly, A1 and A2 noted that wolf tooth marks were harder to process, observing that the 3D models obtained from this carnivore presented weaker topographical variations and were thus harder to analyse. Dog tooth marks on the other hand were noted as much deeper, easier to recognise and therefore digitisation procedures were easier overall. While the variations between these marks may be due to a number of variables dependent on the samples used, these go beyond the scope of the present study and are pending further future research.

#### 3.1.1. Raw measurement calculations

**Fig 3:**
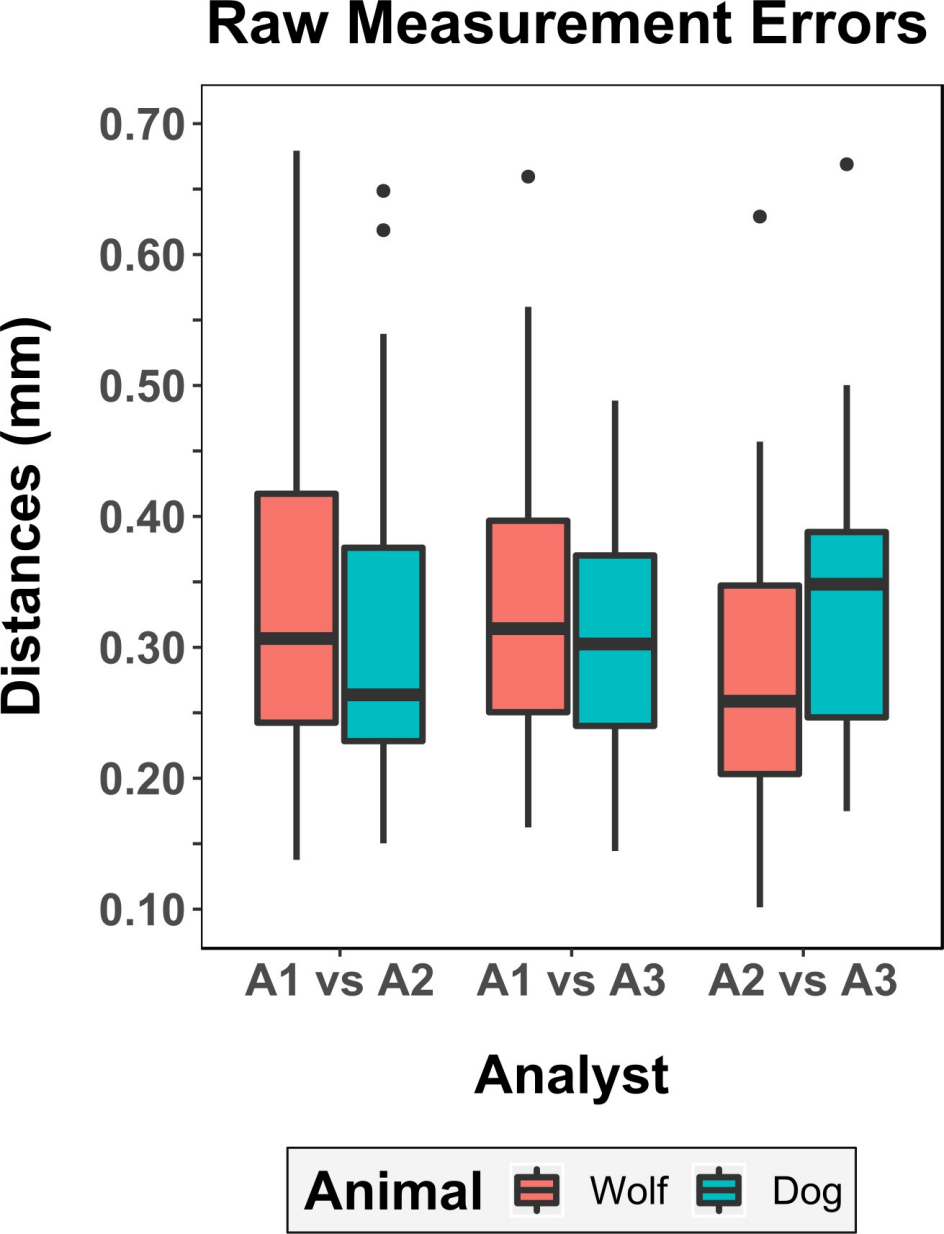
Boxplot representing the distribution of metric errors according to the analyst placing landmarks and the animal being studied.

Raw errors obtained directly from the 3D models indicate an error range of 1.95 mm across the entire data set (Table 1: Min error documented = 0.095 mm, Max = 2.045 mm). Mean and Median errors for all individuals were reported at 0.343 mm and 0.303 mm respectively, with a NMAD of 0.119 mm and square root of the BWMV at 0.121 mm. In all cases, no significant differences were detected when comparing the animal being processed by each analyst (Fig 3: Chi^2^ = 0.027, *p* = 0.8682). Nevertheless, in accordance with the personal observations by A1 and A2, errors calculated on wolf tooth mark samples still present a slightly more varied distribution as opposed to dogs (Fig 3: Wolf Skewness = 4.28, Dog Skewness = 1.61). This indicates that wolves may be harder to process.

**Table 1:** Overall documented metric errors according to the landmarks included in the study and the animal being analysed. BWMV values are reported as the square root of the BWMV. All measurements are recorded in mm. For frame of reference; the studied pits range from 1.176 to 4.448mm in total length and 0.8382 to 3.5128mm in total width.

		Min	Sample Mean	Standard Deviation	Median	NMAD	BWMV	Max
LM 1–17	Absolute	**0.095**	0.343	0.227	**0.303**	0.119	0.121	**2.045**
	Wolf	**0.101**	0.363	0.291	**0.305**	0.128	0.130	**2.045**
	Dog	**0.095**	0.322	0.134	**0.303**	0.109	0.113	**0.966**
LM 1–13	Absolute	**0.048**	0.172	0.084	**0.146**	0.077	0.070	**0.464**
	Wolf	**0.053**	0.172	0.090	**0.143**	0.075	0.084	**0.448**
	Dog	**0.048**	0.171	0.078	**0.158**	0.067	0.070	**0.464**
LM 1–5	Absolute	**0.002**	0.172	0.107	**0.139**	0.083	0.092	**0.586**
	Wolf	**0.002**	0.177	0.120	**0.139**	0.081	0.097	**0.586**
	Dog	**0.009**	0.166	0.094	**0.140**	0.085	0.087	**0.509**

When separating samples according to the observer (Fig 3), a tendency can be observed for larger errors to be recorded when comparisons include the least experienced individuals, nevertheless, kruskal-wallis testing indicates no significant differences (Chi^2^ = 0.98, *p* = 0.61).

Further experimentation with the number of landmarks included reveal that as LM14-17 are removed, error calculations drop to less than half (Table 1: Median = 0.146 mm, NMAD = 0.077 mm, BWMV = 0.070 mm). Likewise, when only considering LM1-5, errors documented on wolf and dog samples are considered the most equivalent (Chi^2^ = 0.0018, *p* = 0.9658). Moreover, when considering each of the LMs separately (Table 2), LM14 presents the highest recorded error among analysts (Median = 0.352 mm, NMAD = 0.292 mm, BWMV = 0.175 mm) while LM5 the lowest (Median = 0.175 mm, NMAD = 0.122 mm, BWMV = 0.075 mm).

**Table 2:** Metric errors according to the landmark being placed. BWMV values are reported as the square root of the BWMV. All measurements are recorded in mm. For frame of reference; studied pits range from 1.176 to 4.448mm in total length and 0.8382 to 3.5128mm in total width.

Landmark	Median	NMAD	BWMV
LM1	0.282	0.191	0.108
LM2	0.279	0.216	0.121
LM3	0.324	0.211	0.125
LM4	0.342	0.229	0.137
LM5	0.175	0.122	0.075
LM6	0.279	0.162	0.103
LM7	0.287	0.176	0.111
LM8	0.315	0.216	0.123
LM9	0.271	0.214	0.116
LM10	0.321	0.233	0.121
LM11	0.288	0.221	0.132
LM12	0.339	0.209	0.135
LM13	0.297	0.172	0.114
LM14	0.352	0.292	0.175
LM15	0.26	0.189	0.122
LM16	0.278	0.203	0.123
LM17	0.315	0.202	0.13

**Fig 4:**
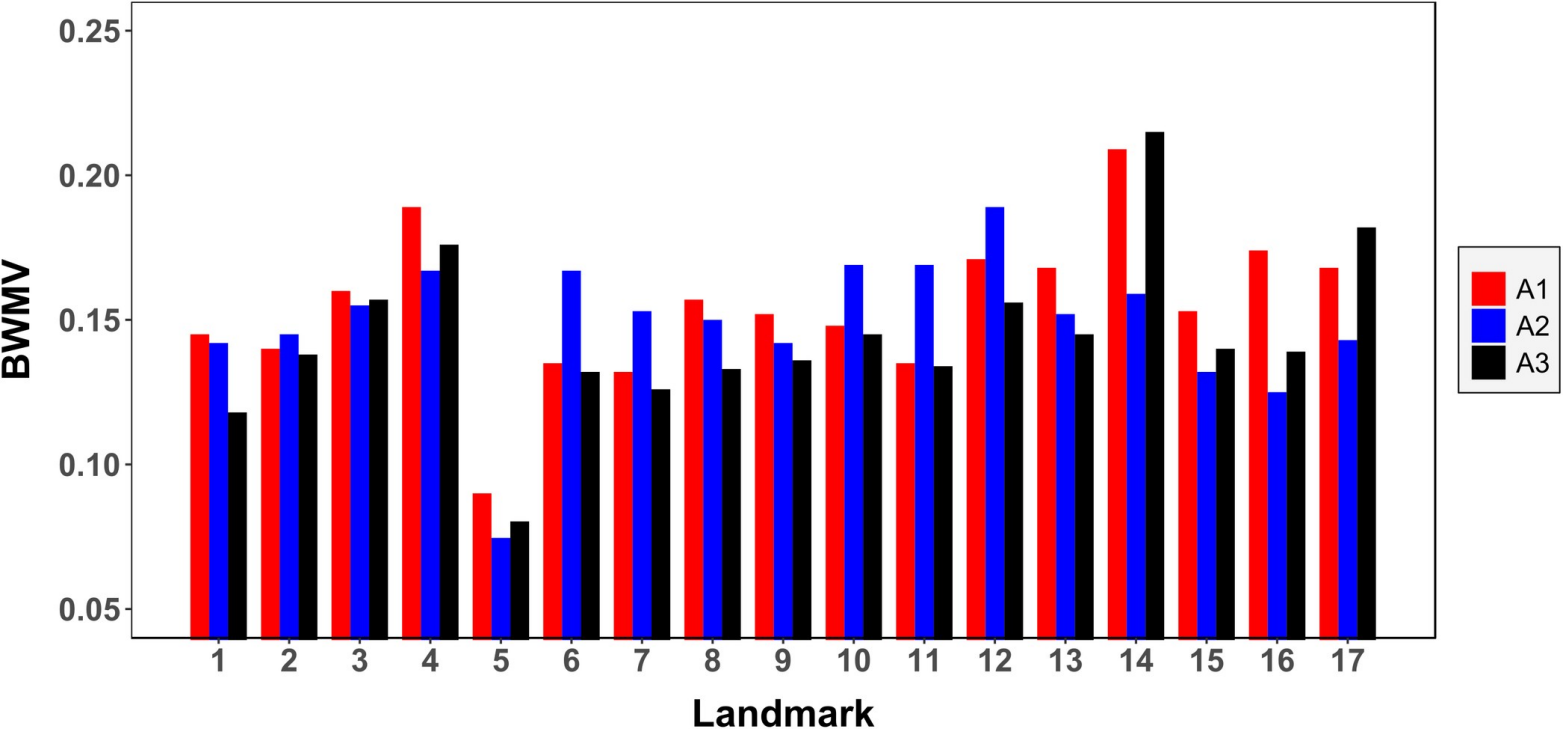
Inter-analyst raw measurement errors for each of the landmarks. Measurements represent the square root of the BWMV of Euclidean distances (mm) from each of the points placed by the analyst to the landmark’s absolute centroid by all three analysts.

When analysing inter-analyst variability according to each individual landmark (Fig 4, S1-S4 Tables of S1 File), errors begin to appear indicating a significant negative correlation between the experience of the analyst and the error produced (Kendall’s τ = -0.03, p = 0.017), proving the error to be significantly lower in analysts of more experience working with GMM than those in the process of learning. Nevertheless, differences in inter-analyst error are only revealed to be significant when considering wolves separately (S3, S4 Tables of S1 File; Chi^2^ = 11.6, *p* = 0.003), while landmark placement appears much more consistent among analysts when analysing dogs (S3, S4 Tables of S1 File; Chi^2^ = 0.36, *p* = 0.8).

#### 3.1.2. Fully superimposed calculations

**Fig 5:**
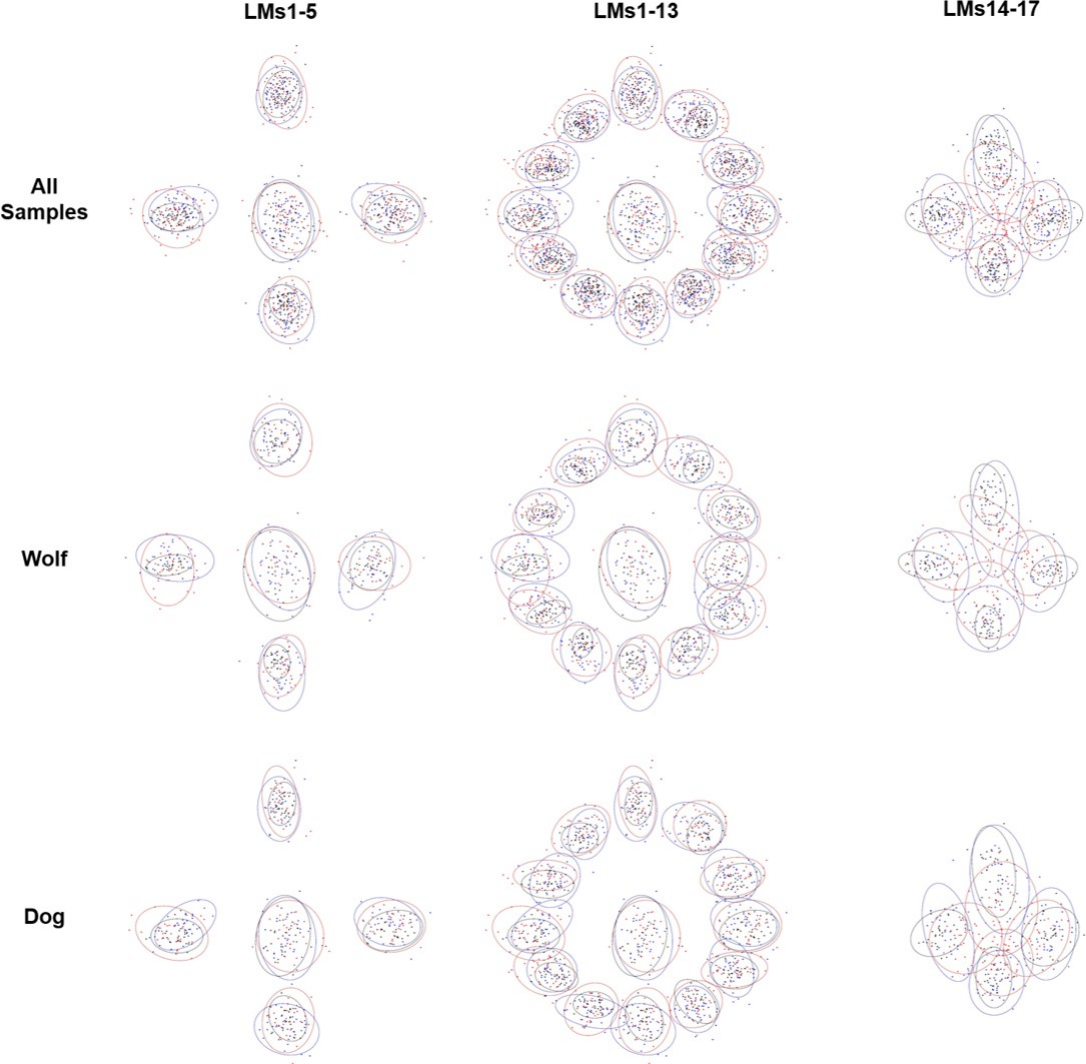
Fully superimposed landmarks in shape space and 95% Confidence ellipses calculated for spatial distributions (A1 = Red, A2 = Blue, A3 = Black).

**Fig 6:**
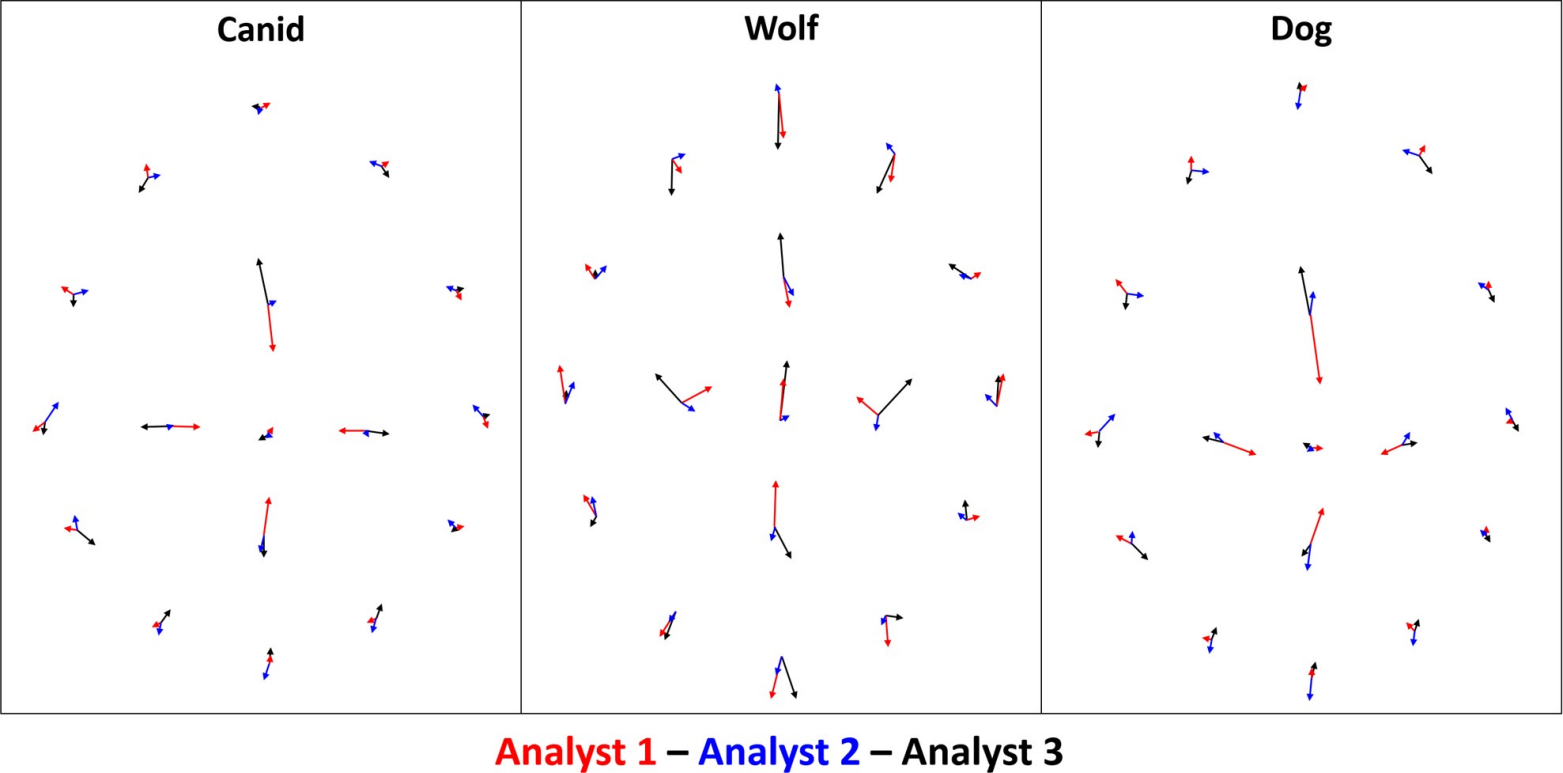
Deviations of landmark centroids calculated from mean configurations in fully superimposed shape space. The origin of each arrow marks the overall centroid for the corresponding landmark in the absolute mean configuration. The end of each arrow marks the position of the same landmark’s centroid according to the analyst.

Fully superimposed coordinates present highly non-Gaussian spatial distributions on all accounts (Shapiro *w* > 0.78, *p* < 2.2e-16), while distribution of landmark centroids reveal interesting differences among the distribution of landmarks according to the analyst (Figs 5 and 6). In most cases, the greatest differences can be detected for LM14-17. Moreover, LM14-17 can clearly be seen to present the highest differences among analysts by observing point cloud distributions (Fig 5) as well as differences in centroid location (Fig 6). Likewise, LM1 to LM5 appear to be the most consistent.

**Fig 7:**
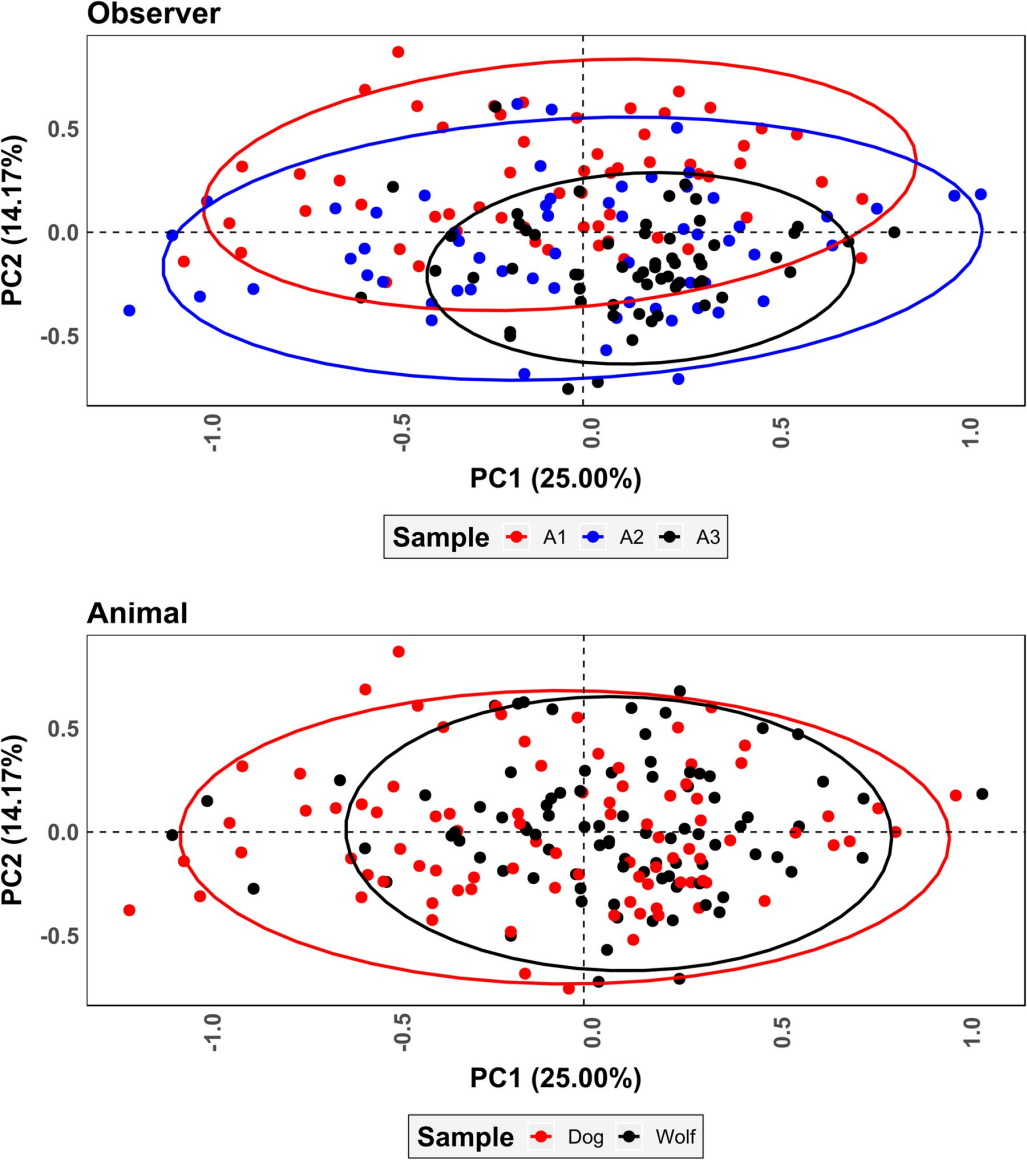
Scatter plot of principal components analysis results for fully superimposed landmark coordinates. Ellipses represent 95% confidence intervals.

PCA results on superimposed coordinate data (Fig 7 and S1 Fig of S1 File) reveal an inhomogeneous (Shapiro *w* = 0.99, *p* = 0.0002) wide dispersal of points with no clustering occurring according to the pit being studied (S1 Fig of S1 File). Clear differences among the analysts can be observed with A1 and A2 occupying a vast area of the represented feature space while A3 presents the least amount of variation (Fig 7). MANOVA calculations obtained from this data indicate significant differences between observers (*p* = 0.001). While significant differences are also detected between wolf and dog samples (*p* = 0.002), the latter *p* values are slightly higher indicating the observer to have a stronger weight over variations detected in PCA than the carnivore.

Procrustes distance calculations (Table 3) reveal a mixture of significant and insignificant differences among the different analysts (Table 4), while comparisons with A1 and A3 show the greatest degrees of variation. These differences are equally evident when comparing carnivore samples separately. Overall distances highlight A3 to be the analyst closest to the absolute mean configuration presenting the least amount of intra-analyst variability (RM = 0.251). Nevertheless, this highlights how analysts with greater experience in GMM are significantly separable from those beginning (Table 3). RMs in general (A1 = 0.403, A2 = 0.357, A3 = 0.251) indicate that a great degree of variation detected within the samples is due to the analyst rather than the true variation produced by the carnivores. This is especially worrying considering how this variation increases according to the experience of the analyst. UPGMA tree topologies additionally reflect this, with absolutely no grouping according to the carnivore under study (S2 Fig of S1 File), and a slight agglomeration of A3 individuals grouped towards the middle of the tree’s branches.

**Table 3:** Median, NMAD and square root of the BWMV values for Procrustes distances in fully superimposed shape space between each of the tooth pits processed by the analysts and the mean configuration.

	Analyst	Median	NMAD	BWMV
Overall	A1	0.230	0.071	0.062
	A2	0.205	0.041	0.051
	A3	0.165	0.028	0.038
Wolf	A1	0.219	0.080	0.067
	A2	0.197	0.047	0.057
	A3	0.155	0.028	0.034
Dog	A1	0.233	0.061	0.054
	A2	0.208	0.044	0.052
	A3	0.171	0.024	0.037

**Table 4:** Kruskal-Wallis Chi^2^ and p-values comparing Procrustes distances in fully superimposed shape space between each of the analysts and the overall mean configuration. Calculations are provided for both carnivores as well as wolf and dog samples separately. Significant p-values under the standard alpha (α) threshold of 0.05 are marked in bold.

		Chi^2^	*p*-Value
Overall	A1 vs A2	1.56	0.21
	A1 vs A3	20.23	**6.9e-06**
	A2 vs A3	16.24	**5.6e-05**
Wolf	A1 vs A2	0.42	0.52
	A1 vs A3	9.00	**0.0027**
	A2 vs A3	7.97	**0.0047**
Dog	A1 vs A2	0.50	0.48
	A1 vs A3	10.29	**0.0013**
	A2 vs A3	7.89	**0.0050**

**Fig 8:**
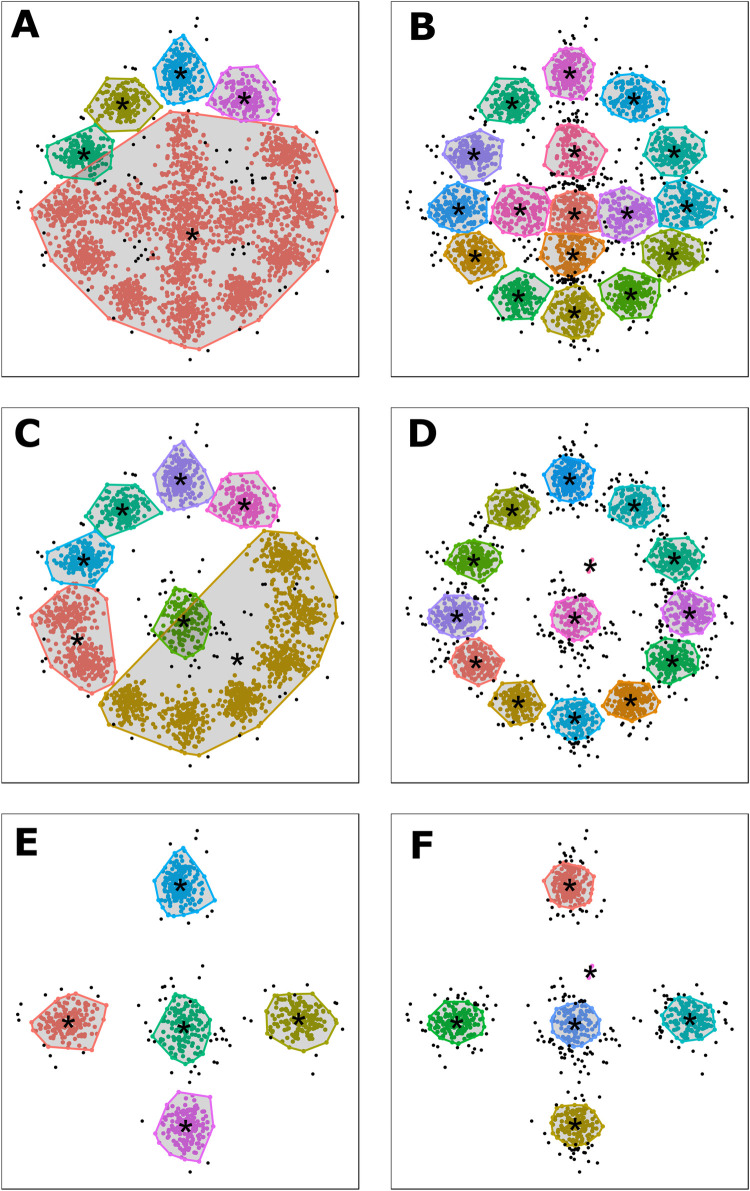
Unsupervised DBSCAN (A, C, E) and MS (B, D, F) pattern recognition results trained on fully superimposed landmark coordinates. Detected clusters are marked by their corresponding colours and calculated convex hulls. Points classified as noise are marked in black. The centroid or point of interest for each cluster is marked by a single black *. (A, B) LM1-17; (C, D) LM1-13; (E, F) LM1-5.

Pattern recognition results classify a high number of points as noise (Fig 8 and S3 Fig & S5 Table of S1 File; DBSCAN = 74, MS = 250), with the least amount of noise detected when only considering LM1-5 (Fig 8E and 8F). Likewise, the highest misclassification rates appear when LM14-17 are included in the models (S5 Table of S1 File; DBSCAN = 2110, MS = 167). Visually, while MS is still able to clearly define the 17 landmarks (Fig 8B), misclassification and noise rates are considerably higher. DBSCAN on the other hand appears much more susceptible to confusion created by density, only clearly identifying points of interest when only LM1-5 are included (Fig 8E). In both cases, pattern recognition helps identify 5 clear points of interest that can be considered key landmarks (Fig 8E and 8F).

#### 3.1.3. Partially superimposed calculations

Partially superimposed coordinates present highly non-Gaussian spatial distributions on all accounts (Shapiro *w* > 0.94, *p* < 1.99e-10). Procrustes distance calculations (Table 5) reveal insignificant differences among all three analysts (Table 6), while comparisons with A1 and A3 show the greatest degrees of variation. This is observed when comparing both carnivore samples together as well as separately (Table 6). Considering each of the analyst’s deviation from the mean reference configuration (Table 5), A3 is seen to present the smallest differences and the greatest consistency when placing landmarks (RM = 0.301). A2 (RM = 0.328) on the other hand is frequently seen separate while A1 presents the least amount of repeatability (RM = 0.383). Considering all three RM scores, a moderate degree of variation is detected within the sample due to the analyst rather than the true variation produced by the carnivore. UPGMA tree topologies additionally reflect this, with no grouping according to the carnivore under study (S4 Fig of S1 File).

**Table 5:** Median, NMAD and square root of the BWMV values for Procrustes distances in partially superimposed shape space between each of the tooth pits processed by the analysts and the mean configuration.

	Analyst	Median	NMAD	BWMV
Overall	A1	1.056	0.375	0.410
	A2	0.977	0.394	0.420
	A3	0.962	0.362	0.329
Wolf	A1	0.976	0.450	0.460
	A2	1.083	0.445	0.383
	A3	0.962	0.417	0.335
Dog	A1	1.040	0.318	0.375
	A2	0.959	0.351	0.447
	A3	0.946	0.382	0.383

**Table 6:** Median, NMAD and square root of the BWMV values for Procrustes distances in partially superimposed shape space between each of the tooth pits processed by the analysts and the mean configuration.

	Analyst	Median	NMAD	BWMV
Overall	A1	1.056	0.375	0.410
	A2	0.977	0.394	0.420
	A3	0.962	0.362	0.329
Wolf	A1	0.976	0.450	0.460
	A2	1.083	0.445	0.383
	A3	0.962	0.417	0.335
Dog	A1	1.040	0.318	0.375
	A2	0.959	0.351	0.447
	A3	0.946	0.382	0.383

**Fig 9:**
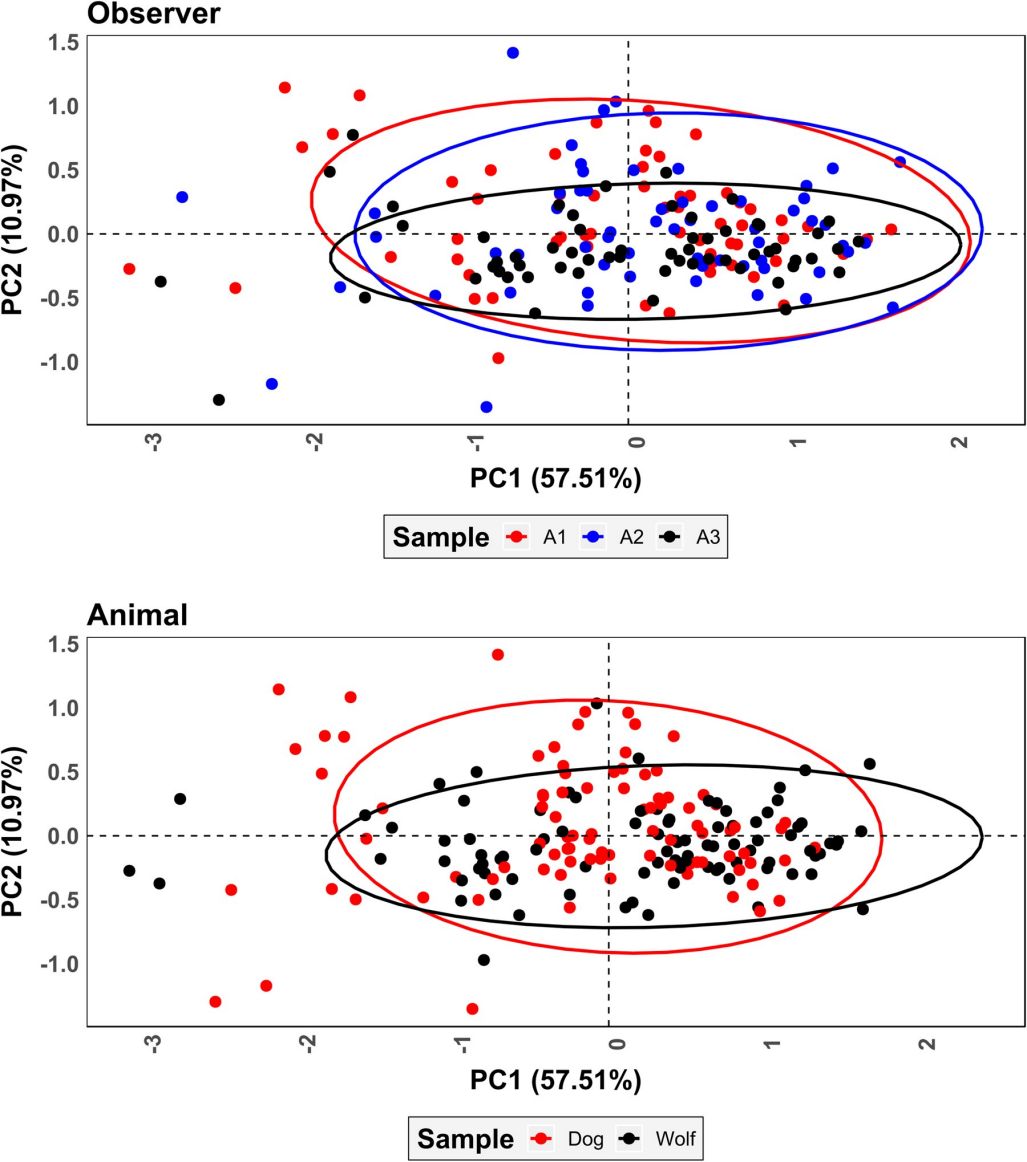
Scatter plot of principal components analysis results for partially superimposed landmark coordinates. Ellipses represent 95% confidence intervals.

PCA results on partially superimposed coordinate data (Fig 9 and S1 Fig of S1 File) yet again reveal an inhomogeneous (Shapiro *w* = 0.90, *p* = 2.2e-16) distribution of points. As opposed to fully superimposed data (Fig 7), however, clustering according to the pit being studied is stronger (S1 Fig of S1 File). At no point, however, is the grouping of points as strong as should be expected, with great variation within the represented feature space being product of the analyst. Once again A3 occupies a much more restricted proportion of feature space, however the difference between distributions is not as extreme. MANOVA calculations indicate equally significant differences between observers as in fully superimposed shape space (*p* = 0.001), however differences between animal samples remain significant regardless of the observer (*p* = 0.001).

### 3.2. Overall evaluation of the 17-landmark model

Comparison of results obtained through raw coordinate data as well as fully and partially superimposed landmarks are sufficient in drawing two main conclusions about the 17 landmark model proposed by Aramendi et al. [55]; (1) experience in GMM is a highly conditioning factor in the amount of error produced within the model and (2) error is observed to increase upon the inclusion of LM14-17.

While the observed error does not necessarily correlate with the type of landmark being used (Kendall’s *τ* = -0.26, *p* = 0.14), Type III landmarks such as LM6-13 tend to present lower degrees of error because their location is influenced by their spatial relationship with Type II LM1-4. If we were to then exclude LM6-13, the weight of this correlation substantially increases (*τ* = 0.45, *p* = 0.09), arguing LM14-17 to be the greatest source of error. This logically agrees with observations made by multiple authors on the value and accuracy of Type III as opposed to Type II landmarks [60, 61, 72].

From a different perspective, while the relationship between experience and repeatability is relatively clear and logical to assume [62–68], upon analysing RMs alone no reliable *p* values can be used to quantify this correlation for this case study (*τ* = -1). When including the metric errors and overall distances between configurations, however, correlations can be successfully calculated supporting the aforementioned conclusions (Kendall’s *τ* = -0.03, p = 0.017).

Further in depth analysis of overall results presents A3 to be the most consistent in placing landmarks regardless of the sample. Nevertheless, other underlying factors may be conditioning some of the results that are harder to quantify. For example, A1 and A2 took little time when placing landmarks (ca. < 1 min), taking a total of approximately 3 hours to process the entire sample. A3 on the other hand took longer processing each pit (ca. > 1 min), and took over twice as long to process the entire sample (approximately 6.5 hours). While the time taken to process a model might not be the most important variable, here it can be argued that elements of an observer’s personality are likely to take some effect in the results, as more meticulous individuals can be seen to take longer and may handle the sample differently. These observations, however, are more subjective and harder to model, stressing the need for analysts to take exceptional care when processing each individual pit so as to avoid these types of error.

Observations regarding the “*Pinocchio Effect*” present notable differences between fully superimposed, partially superimposed and raw data. With regards to geometric morphometric data, PCA appears to be the test where differences become most notable (S1 Fig of S1 File), with drastic changes to feature space appearing when including the scaling procedure in GPA.

Finally, considering the vast amount of data produced in this study, deductions can be made supporting the decision of removing LM14-17 from future analyses so as to reduce statistical errors. On the other hand, while LM6-13 do not produce significant errors in analyses, under the premise of reducing as much statistical noise as possible that may be dependent on the analyst, we have decided to remove all Type III LMs from the rest of this study as well. According to this decision, the definitive fixed Type II landmarks that will be used in our final model will be LM1-5.

### 3.3. Landmark model comparisons

**Fig 10:**
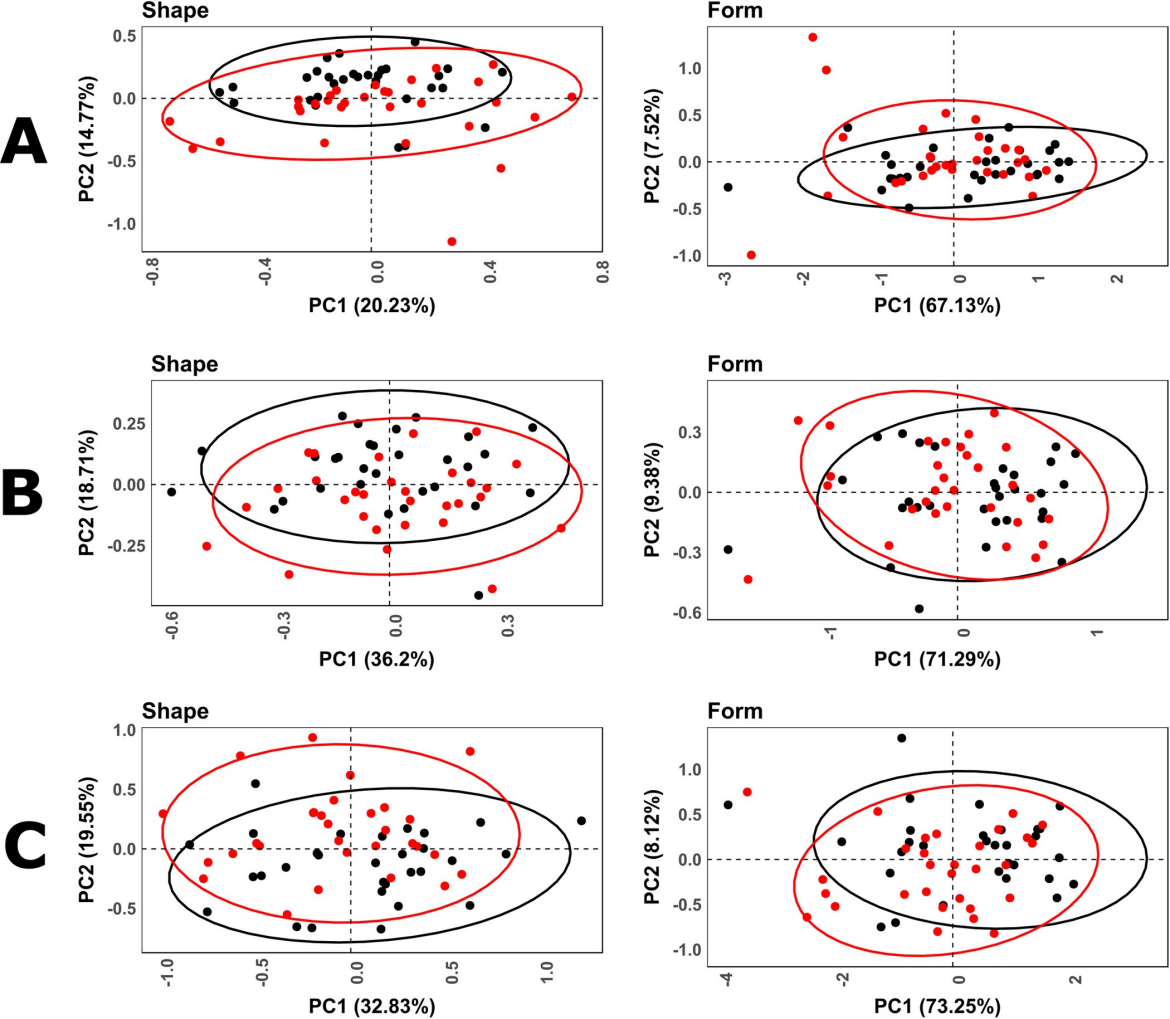
Scatter plots for principal components analysis results comparing different landmark configurations for (Red) dog and (Black) wolf tooth pits. (A) 17-Landmark model in (Left) shape and (Right) form. (B) 5-Landmark model in (Left) shape and (Right) form. (C) 30-landmark model in (Left) shape and (Right) form. Ellipses represent 95% confidence intervals.

**Fig 11:**
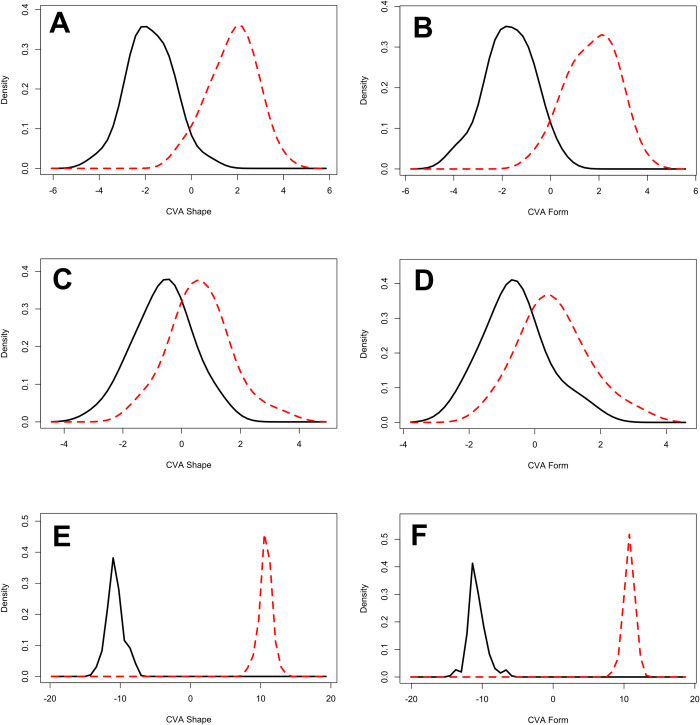
Distribution density plots for Canonical Variance Analyses in (A, C, E) shape and (B, D, F) form comparing (Red) dog and (Black) wolf tooth pits. (A, B) 17-landmark model. (C, D) 5-Landmark model. (E, F) 30-Landmark model.

PCAs (Fig 10A) obtained by the original 17-Landmark model present a non-polarized morphospace with relatively high overlapping of wolf and dog samples in both shape and form space. The defined feature space described by each of these PCAs presents a high number of dimensions in both shape (ℝ^44^) and form (ℝ^51^), representing low overall variability in the first 2 dimensions (shape = 35.39%, form = 74.65%). The first 10 dimensions represent a much higher cumulative proportion of variance (shape = 83.78%, form = 94.39%), with MANOVA results highlighting significant statistical differences between carnivore tooth samples in shape (*p* = 0.001), but not in form (*p* = 0.059). CVA results obtained from this model present a much clearer separation between samples (Fig 11A and 11B), with both significant Procrustes (D = 0.072, *p* = 0.002) as well as Mahalanobis distances (D = 3.55, *p* < 0.0001).

Removal of Type III landmarks with the 5-Landmark model presents a slight increase in overlapping in PCA (Fig 10B) of carnivore samples in a reduced number of dimensions for both shape (ℝ^8^, PC1&2 = 55.50%) and form (ℝ^15^, PC1&2 = 80.67%). CVA additionally presents a significant reduction in both Procrustes (D = 0.064, *p* = 0.04) and Mahalanobis (D = 1.26, *p* = 0.009) distances with high overlapping across graphs (Fig 11C and 11D). Likewise, MANOVA tests indicate differentiation between carnivores to be less clear when using only LM1-5 (shape *p* = 0.029, form *p* = 0.039).

Of the 6 semilandmark models tried and tested, immediate improvements in results were noted with the model employing a 5x5 net of semilandmarks with the 5 fixed Type II landmarks (30 in total, S1 Appendix). PCA results (Fig 10C) display similar graphical results to the original 17-landmark model, with a relatively highly overlapping non-polarized morphospace. Nevertheless, inclusion of semilandmarks display a clear increase in the cumulative degree of variation represented by each graph in both shape (ℝ^59^, PC1&2 = 53.06%, PC1-10 = 92.60%) and form (ℝ^60^, PC1&2 = 81.38%, PC1-10 = 97.91%). These results directly indicate that semilandmark based models are able to increase the chance of finding structure within the data by minimizing bending energy and thus reducing noise [72]. This in turn increases the possibility of finding statistical separation between the samples. These results are also seen through CVA results where clear separations between wolf and dog samples are noticeable (Fig 11E and 11F), with equally as significant Mahalanobis (D = 5.88, *p* < 0.0001) and Procrustes (D = 0.067, *p* = 0.012) distance values. While CVA results are likely to be overestimate separation considering sample size and the increased number of variables when including semilandmarks [102, 103], other statistical tests such as those obtained through MANOVA are also able to highlight significant differences between samples in both shape (*p* = 0.004) and form (*p* = 0.007).

PCA results do not vary to a significant degree upon increasing the number of semilandmarks, with the total accumulative variance increasing by only 0.002% across the first 10 PC scores using 36 semilandmarks. Beyond 49 semilandmarks, percentage of represented variance even begins to decrease and MANOVA results also display poorer separation between samples. Likewise, CVA results on the other hand deteriorate with the inclusion of more semilandmarks, with less clear differences between groups. This is to be expected when considering the sample sizes available, and thus number of semilandmarks should be reduced [102, 103]. This concludes that the optimal statistical model for discerning between carnivore agencies remains to be the 30-landmark model presented here (S1 Appendix).

## 4. Discussion and conclusions

Throughout the years, experimentation has become a fundamental component in archaeological and palaeontological research. Needless to say, with the ever-growing realisation of the impact true analogy has on experimental results, creating parallelisms with the fossil record is often difficult. Product of this is an increasing importance in the availability of experimental samples. This is especially apparent in geographic regions where the present day wild fauna is drastically different to that which existed over 10,000 years ago. In areas such as Europe, access to wild carnivores such as hyenas, lions and leopards is particularly difficult, highlighting the need for collaborative efforts in order to study the archaeological/palaeontological register effectively.

In archaeology/palaeontology, a possible response to this situation has been the collection and pooling of morphometric datasets via online resources [104, 105 *inter alia*], while some datasets are available through supplementary materials [e.g. 56–59]. Nevertheless, pooling of data from different sources is often conflictive, and induced errors are likely to increment if data collection is not strictly controlled [67]. Moreover, in many cases making 3D models readily available often requires a certain degree of computational costs and obtaining permissions to share these files can often be problematic. This latter point is unfortunately due to the additional issue of competitivity amongst many research teams, making scientific progress much more politically, rather than empirically, oriented.

In fields of data science, the sharing of information can be considered indispensable, contributing to some of the most important advances in applied sciences. This is especially relevant when confronting issues of generalisation in predictive modelling [106]. With the advent of *Domain Adaptation* in computational learning [106, 107: pg. 526–531], the development of artificially intelligent Transfer Learning [108, 109], and the creation of One-Shot/Zero-Shot models [110–113], new possibilities for collaborative efforts in data science are now available to solve many research questions. Similar significant advances through *Multimodal* and *Federated Optimization* strategies present new possibilities for the collaborative integration of different datasets [114–116]. Sharing information and training classification models over multiple datasets in this manner presents the distinct advantage of protecting components of data privacy and overcoming issues presented by dataset centralisation [117].

Through more open-minded collaborations among teams, and greater efforts to make these learning strategies available, it may be possible for archaeological and palaeontological researchers to obtain similar success rates to other fields of science [118–120].

Nevertheless, in order for this to happen, careful attention must be paid to the statistical quality of the datasets being used. While tooth pit analyses have been able to reach > 95% classification using GMMs [58, 59, 121], each of these studies have the distinct advantage of landmark data being collected by a single experienced individual [personal communication and direct participation]. Furthermore, while the original 17-landmark model was able to successfully draw conclusions from each of these studies (with the inclusion of [58, 59, 121, 122]), the integration of data produced here may increase the precision of these results. The results presented within this research thus represent a means of removing as much analyst-induced subjectivity as possible in the study of carnivore tooth pits. This is achieved through replacing weak landmark points with more precise computational semilandmarks. Nevertheless, as seen here, LM1-5 still present an important margin of error that must be confronted.

It is common knowledge that in-person training is the most valuable tool for obtaining optimal results in any task. Such an observation has been proven true not only for geometric morphometric research [121], but is also applicable to evolutionary theory [123, 124]. Likewise, the descriptive quality of any metric model is fundamental in its reproducibility. Under this premise, so as to ensure reproducibility and accuracy, a visual guide to the proposed methodological approach has been provided and is available at https://vimeo.com/409256777. This is further accompanied by a detailed description of the fixed landmarks (LM1-5) employed in this study (S1 Appendix), and with graphical representation in Fig 2D. Needless to say, on a practical note the time and care taken to process a 3D model is clearly an important component to consider. Although subjective, personal observations of the different analysts processing the models revealed clear differences between some individuals based on how thorough they were when handling the 3D models. These differences became increasingly obvious as time went by in each work session. While experience is a clear variable of importance, we strongly advise that even the most experienced observers carry out digitisation procedures as meticulously as possible over numerous digitisation sessions and taking full advantage of the tools in most 3D modelling software (e.g. measurement, zoom, pan, and rotating tools). While it is true that repeatability measures are likely to be higher in computerised procedures [66, 69], which is one of the clearest advantages to the methodological approach presented here, work ethic is still a valuable variable that needs to be controlled at all times.

Focusing specifically on the present study, human-induced landmark errors of the new proposed model can be conclusively defined at 0.139 +/- 0.092 ∈ {0.002:0.586} mm (Median +/- √BWMV ∈ {Min:Max}). This presents a total drop in error of 164 μm from the original model which is extrapolated mainly from the statistically noisy landmarks LM14-17. As can be seen by the error range intervals, differences between samples are highly skewed, supporting our use for a robust statistical approach in defining the final model [83]. While the impact this margin of error has on statistical results is arguably not likely to affect the model’s ability to separate between carnivore samples (as seen through MANOVA results), it is important to highlight the power that *form* has over *shape* in geometric morphometric analysis [47].

While Procrustes analyses are a valuable tool for most applied geometric applications [35, 65, 125, 126], it has been seen throughout our results how full Procrustes superimposition produces a notable statistical distortion that cannot be ignored in taphonomic GMM. This is direct evidence of the power the “*Pinocchio effect*” can have on geometric morphometric data (Current study: S1 Fig of S1 File; [65, 82]). Nevertheless, recent research into the power of computational learning algorithms highlight the value of *form* data in the predictive modelling of tooth pits [59]. This supports the removal of scaling processes for tooth pit classification tasks, additionally providing an interesting reflection on *form* and *shape* theory in modern morphometrics [127]. While it is also arguable that the increased presence of a *Pinocchio effect* is product of the microscopic scale being used [64, 66, 70], we insist on our recommendations that *form* data be used when working with decentralized pooled data for classification tasks on tooth pits.

Finally, the variability present in canid tooth marks is of increasing interest, considering the statistical noise they are frequently noted to produce [28, 56–59]. As seen here both qualitatively and quantitatively, wolf tooth marks in general are harder to process and frequently produce an increase in error (Deviations from absolute error = {+4 μm : +14 μm}, Table 1). This is primarily due to their more superficial nature and great variability which is an interesting topic to note and should be explored in detail in the future [58], till then, caution is certainly advised when working with this animal.

In conclusion, this study presents a detailed revision of the current methodological approach available for discerning carnivore agencies via tooth pit morphology. Through this methodological update, we present a means of reducing human induced error in taphonomic geometric morphometric data collection; facilitating the pooling of inter-institutional datasets for the training of classification models. While the theoretical and statistical reflections presented here are extensive, if these protocols are strictly followed and their true implications considered, tooth pit morphological analyses can have a promising future in applied scientific research. Under this premise, we predict lines of investigation of this type will be an encouraging development relevant in palaeontological, archaeological, forensic and even modern-day ecological sciences.

## Supporting information

S1 Appendix (PDF)

S1 File (PDF)
