# Supplementary material for: Obtaining new resolutions in carnivore tooth pit morphological analyses: A methodological update for digital taphonomy
Source: PLoS One. 2020 Oct 8;15(10):e0240328. doi: 10.1371/journal.pone.0240328 (PMC7544140; doi:10.1371/journal.pone.0240328)
Supplement: S1 Appendix — (PDF) [file pone.0240328.s001.pdf]

Obtaining New Resolutions in Carnivore Tooth Pit Morphological Analyses: A Methodological Update for Digital Taphonomy

Supplementary Appendix – Courtenay et al.

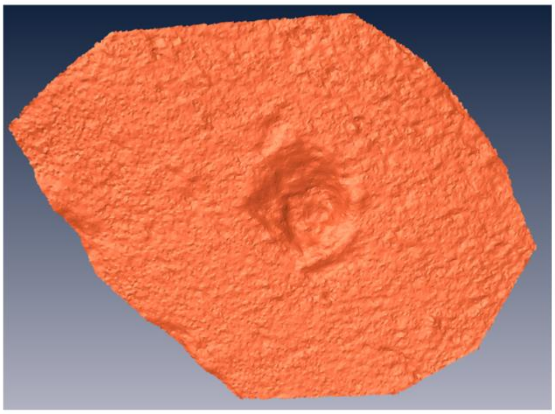

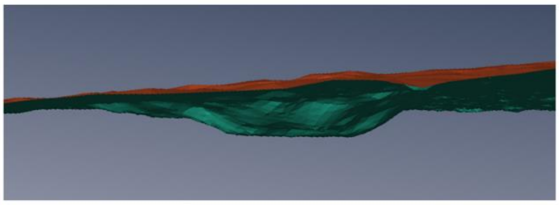

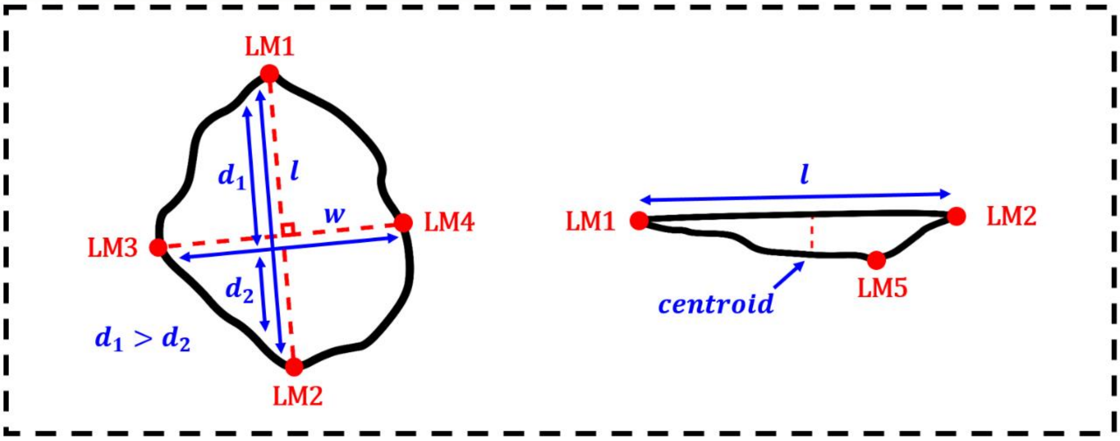

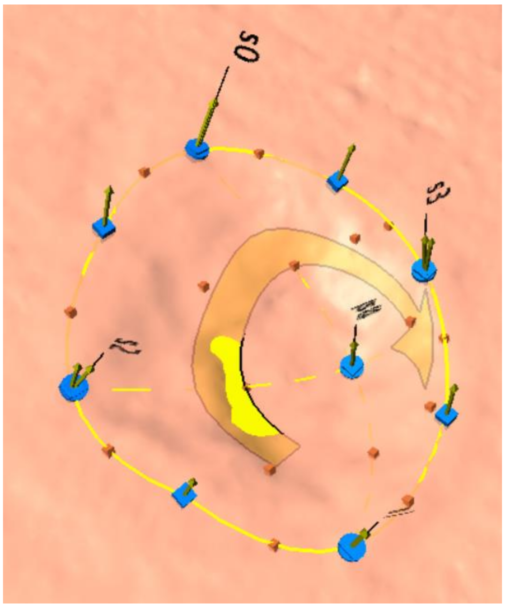

Descriptions accompanying figures can be found in the text below:

LM1 and LM2 mark the maximal length (*l*) of each pit. For the correct orientation of the pit, LM1 is to be considered the point along the maximum length furthest away (d_1) from the perpendicular axis marking the maximum width (*w*). LM2 is thus the point closest to said perpendicular axis (d_2).

LM3 and LM4 marks the maximal width (*w*) of each pit, perpendicular to the axis defined by LM1 and LM2. It is important to ensure that the maximum width is metrically measured so as to ensure precision. LM3 is thus the point that marks the left-most extremity of this perpendicular axis, while LM4 is positioned to the right.

LM5 is the deepest most point of the pit. It is important to highlight that this point is not necessarily the centroid, and should be positioned taking advantage of 3D tools available.

LM6-30 (small orange points shown in the figure above) are calculated and positioned by the computer, using the Landmark Editor v.3.0.0.6 software. For this, the patch tool is used to isolate only the pit in the 3D model. So as to ensure that the orientation of the semilandmarks are comparable with those used within this study, using the “Flip” and “Rotate” options in the “Edit Primitives” window of the “View” dropdown menu, orientate the patch so that the arrow is pointing towards LM2 (the concave shape of the curved arrow should be facing towards LM1). In the same “Edit Primitives” window, select a 5x5 semilandmark mesh.

NOTE: In Landmark Editor v.3.0.0.6. landmarks are numbered beginning with 0. E.g. LM1 = LM0

The final instructional video can be accessed via the following link:

https://vimeo.com/409256777
