# Supplementary material for: Obtaining new resolutions in carnivore tooth pit morphological analyses: A methodological update for digital taphonomy
Source: PLoS One. 2020 Oct 8;15(10):e0240328. doi: 10.1371/journal.pone.0240328 (PMC7544140; doi:10.1371/journal.pone.0240328)
Supplement: S1 File — (PDF) [file pone.0240328.s002.pdf]

Obtaining New Resolutions in Carnivore Tooth Pit Morphological Analyses: A Methodological Update for Digital Taphonomy

Lloyd A. Courtenay, Darío Herranz-Rodrigo, Rosa Huguet, Miguel-Ángel Maté-González, Diego González-Aguilera, José Yravedra.

Supplementary Materials

INDEX

Table S1 – Page 2
Table S2 – Page 3
Table S3 – Page 4
Table S4 – Page 5
Figure S1 – Page 6
Figure S2 – Page 7
Table S5 – Page 8
Figure S3 – Page 9
Figure S4 – Page 10

Landmark	Absolute	A1	A2	A3
LM1	0.138	0.145	0.142	0.118
LM2	0.138	0.14	0.145	0.138
LM3	0.158	0.16	0.155	0.157
LM4	0.177	0.189	0.167	0.176
LM5	0.0828	0.09	0.0746	0.0803
LM6	0.141	0.135	0.167	0.132
LM7	0.133	0.132	0.153	0.126
LM8	0.148	0.157	0.15	0.133
LM9	0.145	0.152	0.142	0.136
LM10	0.159	0.148	0.169	0.145
LM11	0.139	0.135	0.169	0.134
LM12	0.169	0.171	0.189	0.156
LM13	0.153	0.168	0.152	0.145
LM14	0.194	0.209	0.159	0.215
LM15	0.142	0.153	0.132	0.14
LM16	0.141	0.174	0.125	0.139
LM17	0.158	0.168	0.143	0.182

Table S1 – Table presenting the median values for central tendency of raw metric error values (mm) obtained for inter-observer analyses (represented graphically in Figure 4). Non-parametric summary statistics are obtained from the distances calculated between each point and the landmark's absolute centroid. For frame of reference; studied pits range from 1.176 to 4.448mm in total length and 0.8382 to 3.5128mm in total width.

	Absolute		A1		A2		A3	
Landmark	NMAD	BWMV	NMAD	BWMV	NMAD	BWMV	NMAD	BWMV
LM1	0.105	0.057	0.108	0.107	0.104	0.096	0.103	0.092
LM2	0.105	0.062	0.106	0.118	0.121	0.127	0.102	0.082
LM3	0.116	0.068	0.120	0.125	0.142	0.135	0.089	0.097
LM4	0.107	0.069	0.109	0.126	0.103	0.107	0.097	0.122
LM5	0.051	0.034	0.048	0.053	0.049	0.056	0.054	0.064
LM6	0.085	0.051	0.084	0.090	0.084	0.092	0.070	0.079
LM7	0.088	0.058	0.094	0.101	0.100	0.105	0.092	0.097
LM8	0.098	0.062	0.102	0.109	0.134	0.120	0.071	0.089
LM9	0.103	0.060	0.096	0.112	0.121	0.120	0.084	0.077
LM10	0.104	0.058	0.086	0.097	0.097	0.090	0.103	0.107
LM11	0.091	0.062	0.079	0.091	0.113	0.110	0.089	0.109
LM12	0.107	0.070	0.133	0.152	0.095	0.108	0.094	0.111
LM13	0.103	0.061	0.118	0.116	0.099	0.113	0.094	0.089
LM14	0.134	0.080	0.125	0.153	0.120	0.121	0.152	0.131
LM15	0.097	0.060	0.104	0.115	0.094	0.094	0.100	0.100
LM16	0.099	0.061	0.117	0.110	0.087	0.102	0.110	0.099
LM17	0.107	0.063	0.117	0.110	0.099	0.105	0.109	0.109

Table S2 – Table presenting the NMAD and square root of the BWMV values obtained for the raw metric error values (mm) in inter-observer analyses (represented graphically in Figure 4). Non-parametric summary statistics are obtained from the distances calculated between each point and the landmark's absolute centroid. For frame of reference; studied pits range from 1.176 to 4.448mm in total length and 0.8382 to 3.5128mm in total width.

Landmark	Absolute	A1	A2	A3
		Wolf		
LM1	0.130	0.147	0.137	0.116
LM2	0.158	0.180	0.139	0.122
LM3	0.147	0.187	0.147	0.121
LM4	0.192	0.185	0.215	0.174
LM5	0.077	0.084	0.072	0.090
LM6	0.132	0.166	0.151	0.102
LM7	0.127	0.161	0.122	0.108
LM8	0.133	0.165	0.117	0.119
LM9	0.122	0.160	0.110	0.112
LM10	0.162	0.156	0.174	0.142
LM11	0.153	0.124	0.196	0.118
LM12	0.175	0.182	0.213	0.158
LM13	0.152	0.179	0.151	0.131
LM14	0.175	0.175	0.167	0.226
LM15	0.154	0.128	0.138	0.185
LM16	0.137	0.175	0.128	0.146
LM17	0.169	0.144	0.164	0.190
		Dog		
LM1	0.143	0.145	0.161	0.126
LM2	0.137	0.108	0.148	0.145
LM3	0.163	0.146	0.157	0.198
LM4	0.166	0.189	0.133	0.176
LM5	0.089	0.098	0.092	0.079
LM6	0.159	0.127	0.180	0.169
LM7	0.166	0.116	0.184	0.182
LM8	0.160	0.147	0.176	0.171
LM9	0.158	0.143	0.178	0.161
LM10	0.152	0.134	0.164	0.147
LM11	0.138	0.138	0.136	0.142
LM12	0.165	0.126	0.181	0.153
LM13	0.156	0.158	0.167	0.150
LM14	0.203	0.267	0.155	0.213
LM15	0.134	0.165	0.130	0.130
LM16	0.146	0.174	0.124	0.136
LM17	0.152	0.176	0.106	0.166

Table S3 – Table presenting the median values (mm) for central tendency obtained for inter-observer analyses according to the animal being studied. Non-parametric summary statistics are obtained from the distances calculated between each point and the landmark’s absolute centroid for each animal.

Landmark	Absolute NMAD	Absolute BWMV	A1 NMAD	A1 BWMV	A2 NMAD	A2 BWMV	A3 NMAD	A3 BWMV
				Wolf				
LM1	0.079	0.053	0.114	0.133	0.090	0.085	0.057	0.075
LM2	0.123	0.066	0.164	0.135	0.124	0.125	0.085	0.075
LM3	0.091	0.059	0.130	0.140	0.106	0.106	0.070	0.061
LM4	0.147	0.088	0.139	0.163	0.143	0.125	0.130	0.166
LM5	0.049	0.033	0.042	0.046	0.043	0.050	0.069	0.062
LM6	0.092	0.051	0.097	0.104	0.086	0.089	0.054	0.052
LM7	0.073	0.048	0.086	0.102	0.054	0.062	0.043	0.068
LM8	0.075	0.052	0.088	0.106	0.090	0.105	0.051	0.058
LM9	0.086	0.058	0.102	0.119	0.075	0.104	0.071	0.068
LM10	0.096	0.062	0.103	0.121	0.083	0.085	0.110	0.119
LM11	0.108	0.071	0.068	0.076	0.105	0.110	0.076	0.115
LM12	0.129	0.077	0.172	0.165	0.139	0.125	0.120	0.118
LM13	0.100	0.060	0.104	0.118	0.093	0.113	0.086	0.083
LM14	0.131	0.070	0.099	0.091	0.141	0.142	0.143	0.120
LM15	0.115	0.065	0.087	0.110	0.105	0.103	0.121	0.122
LM16	0.105	0.060	0.121	0.100	0.092	0.101	0.118	0.106
LM17	0.103	0.061	0.102	0.098	0.078	0.100	0.113	0.111
				Dog				
LM1	0.112	0.055	0.106	0.087	0.097	0.101	0.126	0.098
LM2	0.101	0.059	0.071	0.079	0.112	0.126	0.097	0.087
LM3	0.143	0.073	0.096	0.107	0.165	0.155	0.128	0.111
LM4	0.081	0.049	0.078	0.086	0.059	0.069	0.080	0.091
LM5	0.059	0.038	0.063	0.067	0.072	0.071	0.050	0.060
LM6	0.089	0.052	0.072	0.079	0.080	0.093	0.096	0.092
LM7	0.123	0.062	0.082	0.097	0.122	0.115	0.115	0.099
LM8	0.117	0.065	0.104	0.108	0.122	0.120	0.111	0.107
LM9	0.119	0.061	0.104	0.105	0.142	0.127	0.058	0.084
LM10	0.107	0.054	0.075	0.087	0.115	0.093	0.105	0.098
LM11	0.087	0.056	0.087	0.092	0.107	0.105	0.075	0.092
LM12	0.089	0.062	0.124	0.142	0.069	0.080	0.087	0.104
LM13	0.107	0.062	0.108	0.109	0.102	0.115	0.107	0.096
LM14	0.145	0.091	0.206	0.210	0.104	0.101	0.174	0.143
LM15	0.084	0.055	0.112	0.119	0.076	0.089	0.082	0.081
LM16	0.100	0.062	0.110	0.120	0.088	0.105	0.088	0.094
LM17	0.105	0.065	0.103	0.115	0.058	0.073	0.117	0.108

Table S4 – Table presenting the NMAD and square root of the BWMV values (mm) obtained for inter-observer analyses according to the animal being studied. Non-parametric summary statistics are obtained from the distances calculated between each point and the landmark's absolute centroid for each animal.

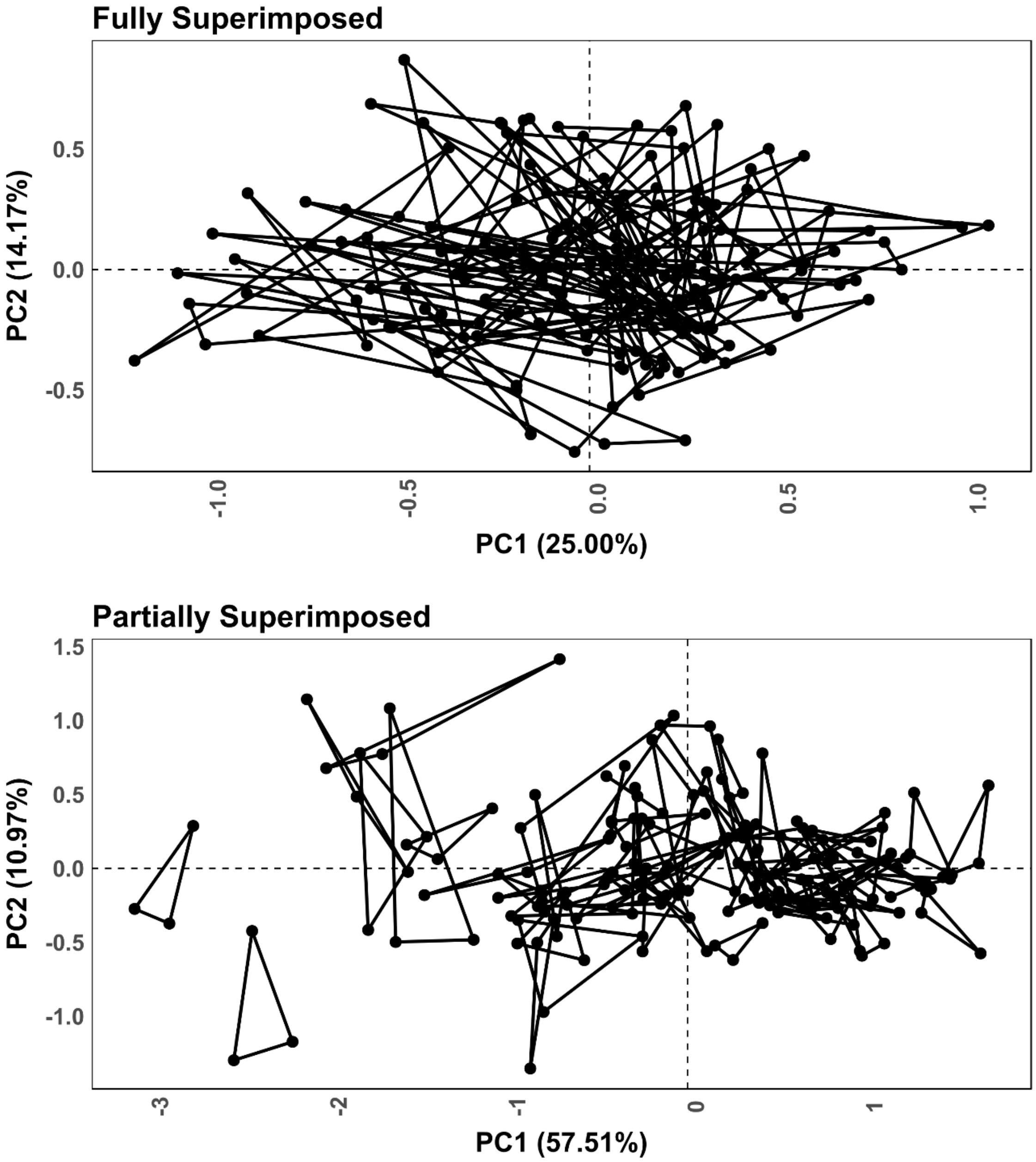

Figure S1 – Scatter plot and convex hulls from PCA comparing both fully (upper panel) and partially (lower panel) superimposed landmark coordinates. Convex hulls in the form of triangles thus connect those pits that have been processed by each of the three different observers.

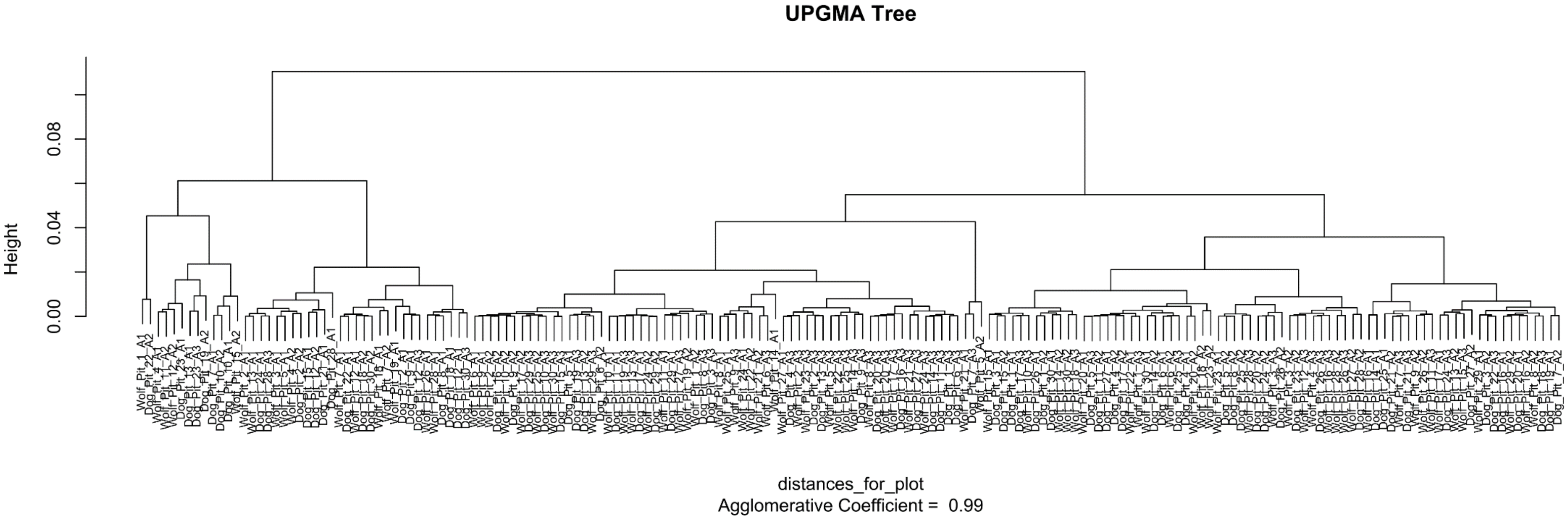

Figure S2 – UPGMA Tree calculated from fully superimposed Procrustes distances across the entire sample.

		LM1-17		LM1-13		LM1-5	
		DBSCAN	MS	DBSCAN	MS	DBSCAN	MS
Total	Number of Clusters	5	17	7	14	5	6
	Correct Class	876	2641	1372	1996	837	706
	Incorrect Class	2110	167	894	13	0	2
	Noise	74	250	74	327	63	192
LM1	Correct Class	172	161	172	151	172	151
	Incorrect Class	3	2	3	0	0	0
	Noise	5	17	5	29	8	29
LM2	Correct Class	0	167	178	151	177	151
	Incorrect Class	178	2	0	1	0	0
	Noise	2	11	2	28	3	29
LM3	Correct Class	2	158	172	147	169	147
	Incorrect Class	172	7	2	1	0	0
	Noise	6	13	6	28	11	33
LM4	Correct Class	0	160	0	144	168	144
	Incorrect Class	176	8	174	2	0	0
	Noise	4	12	6	34	12	36
LM5	Correct Class	180	116	151	113	151	113
	Incorrect Class	0	44	0	2	0	2
	Noise	0	20	29	65	29	65
LM6	Correct Class	175	173	175	162		
	Incorrect Class	0	0	0	0		
	Noise	5	7	5	18		
LM7	Correct Class	171	169	171	160		
	Incorrect Class	5	1	5	1		
	Noise	4	10	4	19		
LM8	Correct Class	0	173	177	165		
	Incorrect Class	177	2	0	1		
	Noise	3	5	3	14		
LM9	Correct Class	0	170	0	163		
	Incorrect Class	177	4	177	3		
	Noise	3	6	3	14		
LM10	Correct Class	176	170	176	156		
	Incorrect Class	2	1	2	0		
	Noise	2	9	2	24		
LM11	Correct Class	0	170	0	160		
	Incorrect Class	177	2	177	1		
	Noise	3	8	3	19		
LM12	Correct Class	0	169	0	158		
	Incorrect Class	177	3	177	1		
	Noise	3	8	3	21		
LM13	Correct Class	0	174	0	166		
	Incorrect Class	177	0	177	0		
	Noise	3	6	3	14		
LM14	Correct Class	0	116				
	Incorrect Class	176	26				
	Noise	4	38				
LM15	Correct Class	0	117				
	Incorrect Class	178	27				
	Noise	2	36				
LM16	Correct Class	0	139				
	Incorrect Class	168	19				
	Noise	12	22				
LM17	Correct Class	0	139				
	Incorrect Class	167	19				
	Noise	13	22				

Table. S5 – Numeric results from unsupervised DBSCAN and MS pattern recognition algorithms trained on fully superimposed landmark coordinates.

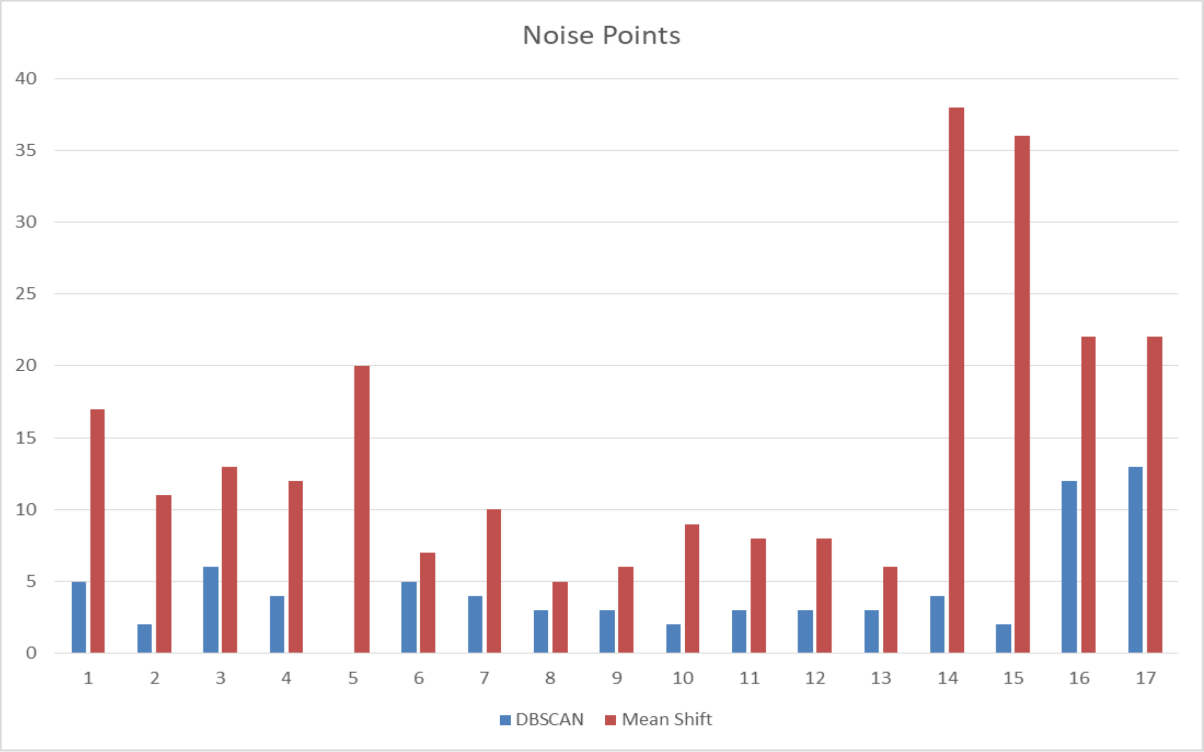

Figure S3 – Bar chart representing the number of points classified as noise for each of the landmarks by each of the pattern recognition algorithms.

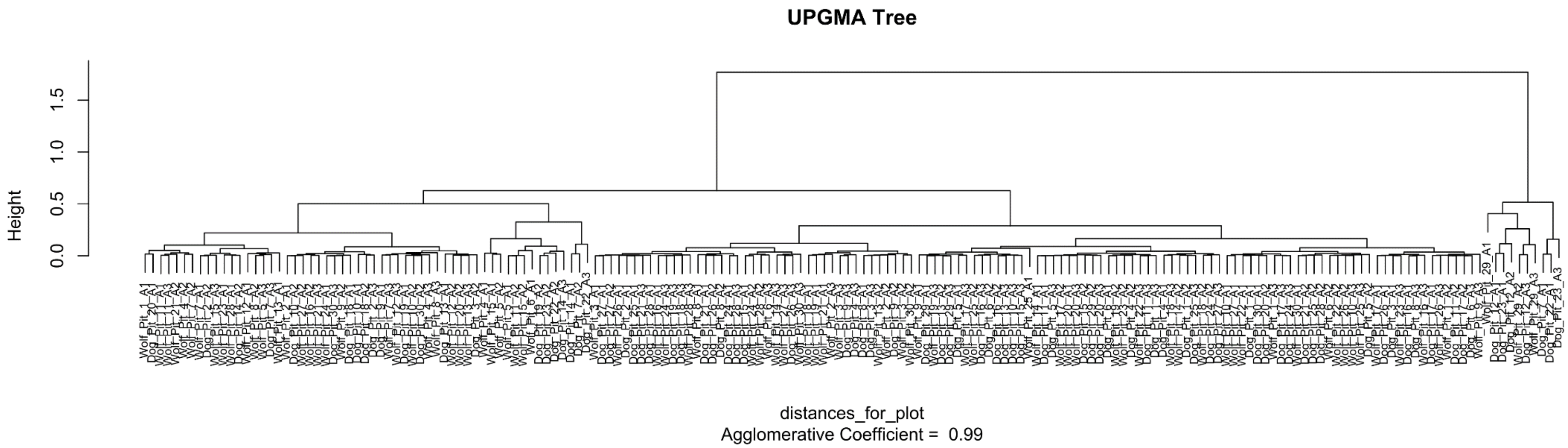

Figure S4 – UPGMA Tree calculated from Procrustes distances across the entire sample in semi-superimposed form space.
